# Complement component 3 haplotypes influence serum complement activity and milk production traits in Chinese Holstein cattle

**DOI:** 10.1371/journal.pone.0268959

**Published:** 2022-06-30

**Authors:** Yonghui Wang, Junyu Zhai, Chunhong Yang, Jingpeng Wang, Yan Sun, Yuhua Li, Zhihua Ju, Jingming Huang, Changfa Wang

**Affiliations:** 1 College of Agronomy, Liaocheng University, Liaocheng City, Shandong Province, P. R. China; 2 Dairy Cattle Research Center, Shandong Academy of Agricultural Science, Jinan City, Shandong Province, P. R. China; Tokai University School of Medicine, JAPAN

## Abstract

Complement component 3 (C3) is the key molecule of the three pathways of complement activation (alternative, classical, and lectin pathways), which are involved in phagocytosis, inflammation, and immunoregulation processes to destroy infectious microorganisms. In this study, three novel single-nucleotide polymorphisms (SNPs) (g.-1293C>G located in the 5′-flanking region, g.56T>C in exon I, and g.7017C>T in exon XII) of the *C3* gene were detected using created restriction site polymerase chain reaction, restriction fragment length polymorphism, and DNA sequencing in 952 cattle from three Chinese breeds. The genotypes and haplotypes were analyzed to investigate the polymorphisms and their possible implications, with particular investigative focus on their associations with serum C3 level, complement hemolytic activity (CH50 and ACH50), and milk production traits. The g.56T>C SNP in exon I affected the serum ACH50 (*P*<0.01) and the milk somatic cell score (SCS) (*P<*0.05), and the g.7017C>T SNP in exon XII significantly affected the serum ACH50 values (*P*<0.01). Moreover, statistical analyses revealed that individuals with genotypic combination CCC/GCC showed significantly lower SCS and the lowest C3 concentration in serum compared with cows with CCC/GTT (*P = 0*.*0007*) and CTT/CTT (*P = 0*.*0021*); the individuals with CCC/CCT had significantly higher ACH50 values than those with CCC/CTC (*P = 0*.*0008*) and CTC/GTC (*P = 0*.*001*); cows with CCT/CTT had higher values of CH50 and 305-day milk yield (*P*>0.05). The C3 expression levels were significantly increased in lung and mammary tissues (*P*<0.05), while significantly decreased in heart, spleen, liver, and kidney tissues in mastitis cows compared with those in healthy animals (*P*<0.01), respectively. Bacterial counts of serum antibacterial activities were also completed to verify the effect of SNPs on resistance to mastitis pathogens. Genetically resistant cows (CCC/GCC) had serum with noticeably higher antibacterial activity against *S*. *aureus* and *E*. *coli* in vitro than the genetically susceptible CCC/GTT cows (*P*<0.05). Results from this study imply that the *C3* gene plays a role in resistance to bacterial infection and that it can be used as a molecular marker for complement activity and traits related to milk production.

## Introduction

Mastitis, an inflammatory disease of the mammary gland that is generally caused by intramammary infections, is the most frequent, complex, and costly disease in dairy cattle [1]. Various bacteria including contagious and environmental bacterial pathogens, such as *Staphylococcus aureus*, *Streptococcus uberis*, and *Escherichia coli*, cause mastitis [2, 3]. The disease causes considerable economic losses due to decreases in the quality and quantity of milk production, increase in the cost of treatment and veterinary services, and early culling [1, 4]. In addition to therapeutic, prophylactic, and management strategies, one promising approach to reduce mastitis is the selection of animals that are resistant to mastitis and the incorporation of this trait into herds. Although genetic variability for mastitis resistance represents only a small proportion of the total variance, it is not negligible and provides an attractive complementary way to improve mastitis resistance in dairy cattle. Thus, strategies for recognizing and describing these genes are integral to the development of marker-assisted selection, which can increase their frequency and effectiveness, leading to improved ability to combat these pathogens.

**Fig 1:**
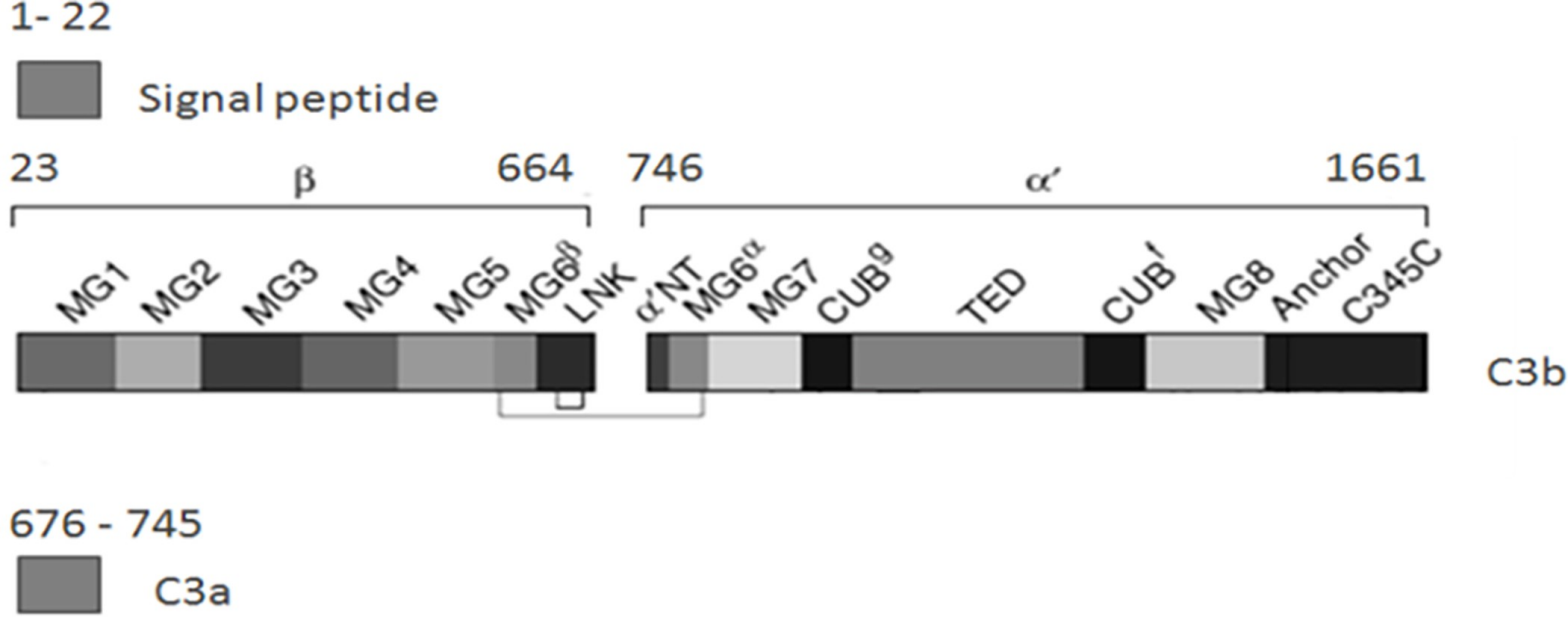
Shows the protein fragment of C3: The signal peptide and the alpha and beta chains, which consist of the C3a and C3b fragment.

Resistance/susceptibility to mastitis is a complex trait, and genes involved in the innate immune response have been indicated as strong candidates [1, 5, 6]. The bovine complement system is present in serum and milk and plays an important part in the defense mechanism of the mammary gland against mastitis [7–9]. Complement component 3 (C3) contributes to overall defense, where it is an integral part of the complement system and a key molecule of the three complement reaction pathways: the classical pathway (triggered by antibodies), the lectin pathway (triggered by lectin), and the alternative pathway (triggered directly on pathogen surfaces). The bovine *C3* gene is located on chromosome 7, and it contains 40 introns and 41 exons encoding 1661 amino acids (Fig 1). The C3 protein, which is a member of the α_2_M family, is a 190 kDa glycoprotein essential for eliciting the complement response. It consists of two polypeptide chains (alpha and beta) held together with a single disulfide bridge. An internal thioester in the alpha chain of C3 is cleaved during complement activation. This mediates covalent attachment of the activated C3b to immune complexes and invading microorganisms, thereby opsonizing the target [10].

Innate immunity is involved in phagocytosis, inflammation, and immunoregulation processes to destroy infectious microorganisms [11, 12]. Second, the study of transcriptome profiling of *Streptococcus uberis*-induced mastitis reveals fundamental differences in immune gene expression of the mammary gland and the primary cell culture model. For example, the *C3* gene is upregulated by 2.36-fold compared with non-infected control groups [6]. Third, Affymetrix DNA microarrays showed that the mRNA abundances of the *C3* gene were strongly and significantly increased (3.8-fold) in mammary epithelial cells (MECs) stimulated by *E*. *coli* [13]. Furthermore, complement hemolytic activity and C3 concentration in milk are higher in mammary gland with mastitis than in healthy mammary gland [14, 15]. Finally, some studies also found a significant association between different genotypes of C3 and milk production in sheep (*P<0*.*01*) [16] and classical complement pathway and alternative complement pathway activity in porcine (*P<*0.05) [17]. However, little information is available about the bovine *C3* single-nucleotide polymorphisms (SNPs) and their associations with infectious bacterial diseases and complement hemolytic activity.

Chinese Holstein Cattle is derived from grading cross breeding and selection between Chinese native cow and purebred Holstein bull introduced in China. The frequency of mastitis in this breed is about 38%–50% [18]. The Luxi Yellow cattle and Bohai Black cattle are two of the representative indigenous bovine (*Bos taurus*) breeds in China, which have been bred as beef and draft dual-purpose cattle for thousands of years because of their disease resistance and ability to endure unfavorable feeding conditions. The aim of the current study was to investigate the SNPs of the *C3* gene and assess their possible associations between SNPs, combined genotypes with SCS, serum C3 concentration, and complement hemolytic activity (ACH50 and CH50) in Chinese Holstein cattle [19].

## Materials and methods

### Ethical statement

All animal protocols were approved by the Animal Welfare & Ethics Committee of Institute of Animal Sciences, Liaocheng University (No. LC2019-1).

### Study animals

The animals used in this study were selected from three common Chinese bovine breeds, including Chinese Holstein (CH; n = 821), Luxi Yellow (LX; n = 106), and Bohai Black (BH; n = 25). Chinese Holstein cattle (4–7 years old and calving numbers one to four) were randomly selected from eight dairy farms in the Jinan and Qingdao Agriculture Development Area, China. Each of the included farms had complete lactation Dairy Herd Improvement (DHI) records. Of the 821 selected individuals, 354 had sufficient serum volume for complement hemolytic activity (CH50 and ACH50) and C3 concentration tests, and samples from these individuals were included in the final analysis. Individuals of the Luxi Yellow and Bohai Black breeds (mean age 14 months) were randomly selected from their original conservation areas. The milk performance parameters (305-day milk yield, somatic cell score (SCS), fat and protein percentage) of Chinese Holstein from 27 sires were provided by the DHI Laboratory of the Dairy Cattle Research Center of Shandong Academy of Agricultural Sciences using a milk composition analyzer (Foss MilkoScan FT 6000, Denmark).

### Chemicals and reagents

All chemicals were obtained from Sigma (Saint Louis, USA) except as noted. The EZgene™ Blood Genomic DNA Miniprep Kit, Platinum Taq DNA polymerase, restriction endonucleases, and associated buffers were obtained from TaKaRa Biotechnology Co., Ltd. (Dalian, China). The Bovine Complement 3 ELISA Kit was obtained from BlueGene (Shanghai, China). Oligonucleotide primers were obtained from Sangon Biological Engineering (Shanghai, China). DNA sequencing was performed by TaKaRa (Dalian, China).

### DNA extraction

Blood samples were obtained from bovine jugular veins and divided into two treatments. In one tube, 10 ml of blood was mixed with 1.6 ml of the anticoagulant acid-citrate-dextrose (0.48% citric acid, 1.32% citrate sodium, 1.47% dextrose) and subsequently stored at -20°C. A phenol: chloroform protocol modified from the methods of [20] was then used to extract genomic DNA. The other one was used to isolate serum (Chinese Holstein samples), which was stored at -70°C. Genomic DNA was extracted by using the EZgene™ Blood Genomic DNA Miniprep Kit. The quantity and quality of DNA were analyzed spectrophotometrically and diluted to 50 ng/μl. All DNA samples were stored at -20°C for subsequent analysis.

### Detection of *C3* gene polymorphisms

A total of 21 primer pairs were designed on the basis of the DNA sequence of bovine *C3* gene (GenBank accession NO. NC_007305.4) by Primer 5.0 software and synthesized by Sangon Biological Engineering Technology to detect the SNPs in the sequences from the 5′region to the 33th exon (Table 1).

**Table 1:** The primers to detect the SNPs.

Primer name	Primer sequences 5’→3’	AT(°C)	SAF (bp)/AR
P1	AAGGTGTCGCAATCAGATAAG	57	1300
	TTCCCTTCACCTAATCTCGTCA		g.-1946/g.-547
P2	GCATGCTTAAAAGTAGACTGA	50.4	265
	CCCAGATTTCTCAATACTGTT		g.-376/ g.-102
E1	ACATTGTCCTTGGATAGTGAACT	56.1	1244
	CTAAGTGGCGTCAATCGTGT		g.-621/ g.623
I1	TTTCGCGTGGGTGTATGTGG	58.6	810
	TGGTGACGGTGCTCAGGTAG		g.330/ g.1139
E2	AAGAGTCGGACACGATTGACGCCAC	64.6	605
	AGGACTCTCGGAGCTGGTGCGG		g.613/ g.1218
E3,4	TTTCCAAATTCTTGCCTCAT	52.7	975
	ACAGTAACCGGGTGTCTATCT		g.1668/g.2642
I4a	GTGCTTGGCGGGTAGTAGGA	52.9	602
	ACATGCCGCAGTTAAGACG		g.3186/ g.3787
I4b	GATTTGAACCCAAGCTGACTG	57.9	757
	CGTACAAGAACCTGCGATGC		g.3520/ g.4276
E5/7	GGCAGATGTGGGCAGTTATG	57.9	1191
	GCTGTTCCGTCCACCTGTT		g.3646/ g.4836
Gap1	GGGAGGCCATACTGAAACGT	52.9	715*
	TCTCACCATGAGGTCGAAGG		g.5053/ g.5767
E11	TACCCACAGGTGTACGTGAC	54.1	533
	CACATCTATGCGTGCAAACTA		g.5854/ g.6386
E12	ATTGGCAGGCAGATTCTTATC	56.6	464
	GGACCATGTAGGTGTAGTAGCG		g.6666/ g.7129
E12,13	GGGGCTTGGATTTCTCATTG	56.5	895
	CTGGCCTTTGGCATTAATCAGTGTG		g.6518/ g.7412
E13	GCCAAGATCCGCTACTACAC	56.5	453
	ACAGAGCCAGAGGCACAAGA		g.7099/ g.7551
E14	TTCATAAGTAGACGCTTGCAC	52.4	1873
	GGGGAAACAGTAGAAACAAT		g.8646/ g.10518
E15	AGAGTCGCTGAGGTTGGTTC	57.9	860
	CGCCGTGATGTTGGAGTGT		g.9006/ g.9865
E20.21	GGTTTGCCATTGCCTTCTCG	61.2	877
	CTCACTTGCCTCACCCTAATCC		g.17433/ g.18309
E22,23	TGTGGCTGTTCGTACACT	49.1	533
	CCTTTCTAGCTCCGTCTT		g.20622/ g.21154
E24	ATCTGTGATAGGAGCGAATA	49.2	494
	GTGCTGTCCAGGTAGTGC		g.21208/ g.21701
E29/31	GGCACGCAAAGACTACGA	54.9	1777
	GGTTCCCATTCCTTACCG		g.29105/ g.30881
E30/33	TCTCAGTGCCTCTACCAAT	54.7	1295
	TCAAGACCACGTTGCTAAT		g.30195/ g.31453

Note; *When we detect the gap of 63 bp in the NCBI record, we found the Chinese Holstein cows have the 114 bp in the location of the gap by the method of sequencing 15 different dairy cattle farms, so the real length is 766 bp. AT = Annealing temperature.

Polymerase chain reaction (PCR) was carried out in a 25 μl reaction mixture containing 50 ng of template DNA, 10 μmol/l of each primer 0.5 μl, 10× buffer 2.5 μl, 50 mmol/l of Mg^2+^ 0.8 μl, 10 μmol/l of dNTP 0.5 μl, and 5U/μl of Taq DNA polymerase 0.4 μl. After an initial denaturation at 94°C for 5 min, the PCR amplification was performed in 35 cycles using the following parameters: denaturation at 94°C for 30 s, annealing at Tm °C (Table 1) for 30 s, and elongation at 72°C for 30 s. The reaction was continued for a final extension at 72°C for 7 min. The PCR products were evaluated using 1% agarose gel electrophoresis by staining with ethidium bromide.

Three new SNPs were found using the DNA pool sequencing method. The SNP (g.56T>C) in the exon I region of the *C3* gene was genotyped by PCR-RFLP method with *Sty I*, and the SNPs (g.-1293C>G and g.7017C>T) in the 5′ region and the exon XII were genotyped by employing created restriction site PCR (CRS-PCR) with primer Ep and Eex12 containing a nucleotide mismatch, respectively, which enable the use of restriction enzymes for discriminating sequence variations [7]. In brief, the third base C is replaced by G from the 3′ end of primer Ep, and the 11th base C is replaced by A from the 5′ end of primer Eex12, which both create a new *Hinf* Ⅰ restriction site (G/ANTC) (see Table 2). Restriction enzyme selections and fragment sizes are given in Table 2. The detection results of allelic variation at the SNP sites were based on the electrophoretic pattern of the restriction enzyme-treated PCR products.

**Table 2:** PCR primers and PCR–RFLP tests for genotyping SNPs detected in the bovine C3 gene.

SNP/location	Primer name	Primer sequences5’→3’	SAF (bp)/AR	Enzyme Name	Genotype: RES (bp)
g.-1293C>G 5’region	Ep	F:TTTGGACGAAGTGCCCTTAA R:GAGAGCTTCCTTGTTCTTGGAG	439 (-1646/-1208)	*Hinf* І	CC:439 CG:439,416,23 GG:416,23
g.56T>C Exon1	Eex1	F:AAAGGCAGCCTCCAACAACC R: GCTAAGTGGCGTCAATCGTGT	651 (-27/623)	*Sty*Ⅰ	TT:509,142 TC:651,509,142 CC:651
g.7017C>T Exon12	Eex12	F:TAACTACCTGAACCTCTCTGTGC R: GGACCATGTAGGTGTAGTAGCG	125 (7005/7129)	*Hinf* І	CC:75,50 CT:75,50,40,10 TT:75,40,10

Abbreviation: SAF = Size of Amplification fragment; AR = Amplification region, RES = size of fragments at the indicated allele after digestion of the PCR product using the respective restriction enzyme; SNP = single nucleotide polymorphism; Underlined nucleotide marks nucleotide mismatches enabling the creation of a restriction enzymes site for discriminating sequence variations.

In Table 2, SAF means the size of amplification fragment, AR means amplification region, RES indicates the size of fragments at the indicated allele after digestion of the PCR product using the respective restriction enzyme, and SNP means single-nucleotide polymorphism; underlined nucleotides indicate mismatches between sequences, enabling the creation of a discrimination site for restriction enzymes.

### Nucleotide sequencing

The PCR products that showed variable patterns in electrophoresis were sequenced directly from two different directions using the ABI PRISM 3730 DNA analyzer (Applied Biosystems, USA) in accordance with the manufacturer’s instructions. The results were sequenced using the DNASTAR 5.0 package (DNASTAR, Inc., USA).

### Classical complement pathway hemolytic titer (CH50) assay

We examined the hemolytic complement activity in 354 individual cattle to assess the in vitro capacity of the complement system in order to evaluate the role of the complement system in host resistance. We followed the established methods of [7, 9, 21] using sensitized rabbit red blood cells (RRBCs) instead of sensitized sheep red blood cells as target cells for measuring the CH50 of bovine. To examine the CH50 of bovine serum, 354 sera from each dairy cattle were diluted with GVB^2+^ at a ratio of 1:75. Different volumes of the diluted sera ranging from 0.4 ml to 1.0 ml were dispensed into 2.0 ml test tubes, and the total volume reached up to 1.3 ml with GVB^2+^. For each tube, 0.2 ml of sensitized RRBC suspension (5×10^8^ cells/ml) was added. Hemolysis control (100%) and a cell blank control were included. The tubes were incubated at 37°C for 60 min with occasional shaking and centrifuged at 1000 g for 5 min. The absorbance of the supernatants at 414 nm (OD_414_) was spectrophotometrically measured [22]. The OD_414_ values, including those of the 100% hemolysis control, were standardized by subtracting the blank absorbance from all samples. Established methods were used to calculate the CH50 values (units/ml) [23, 24].

### Alternative complement pathway hemolytic titer (ACH50) assay

ACH50 assay was performed in accordance with the method of [7]. In general, the levels of complement are expressed in ACH50 titers, which are defined as the reciprocal of the serum dilution causing 50% hemolysis of unsensitized RRBCs. For most experiments, target erythrocytes were drawn from the same giant New Zealand male rabbit and washed three times with EGTA-Mg-GVB. Two buffers were used in the ACH50 activity assay: EGTA-Mg-GVB (pH 7.3, 10 mM EGTA, 4 mM Mg^2+^) with GVB (5 mM sodium barbiturate, 149 mM NaCl, and 0.1% gelatin) and EDTA-GVB (pH 7.3, 10 mM EDTA, 5 mM sodium barbiturate, 149 mM NaCl, and 0.1% gelatin).

### Measurement of C3 protein level in serum

C3 serum concentration in 354 Chinese Holstein cows was measured using the Bovine Complement 3 ELISA Kit (Blue Gene Biotechnology Co., Ltd., Shanghai, China) in accordance with the manufacturer’s instructions. The kit contains a 96-well test plate, standards of known C3 concentrations, wash buffers, a monoclonal anti-C3, a C3-HRP conjugate, and a substrate solution. The serum samples from the Chinese Holstein cattle and standards of known C3 concentrations were loaded into the appropriate well on the antibody-pre-coated microtiter plate; 100 μl of each serum sample or standard per well and 50 μl of the diluted C3-HRP conjugate were added to each well. The plates were covered and incubated for 1 h at 37°C after the solutions were mixed. The wells were washed, and 50 μl of the substrate for the enzyme was added to each well. After incubation at 25°C for 15 min, the wells were washed three times to remove unbound substrates. For each well, 50 μl of Stop Solution was added. Thereafter, the well was mixed properly. The optical density (OD) at 450 nm was measured within 30 min using an ELISA microplate reader. The calibration curve was constructed with OD values, which corresponded to each of the different standard concentrations. This standard curve allowed OD values to be converted to C3 concentrations, expressed in mg/l.

### Serum antibacterial activity of different haplotypic combinations

Two of the most common mastitis-causing pathogens *Staphylococcus aureus* ATCC29213 and *Escherichia coli* ATCC35218 were purchased from the China Institute of Veterinary Drug Control. These pathogens were incubated in Luria–Bertani (LB) broth or mannitol salt medium and allowed to reach a logarithmic growth phase before bacteria were harvested by centrifugation at 5000 g for 20 min at 4°C. Bacterial pellets were then resuspended in sterilized PBS at a density of 10^5^ CFU/ml and used for further analysis.

Results from the haplotypic combination analysis were used to select 12 serum samples from mastitis-resistant individuals (CCC/GCC, H1H5) and 18 samples from mastitis-susceptible individuals (CCC/GTT, H1H8) for examination of antibacterial activity.

Mixtures containing 100 μl of individual sera from the mastitis-resistant (CCC/GCC) or mastitis-susceptible samples (CCC/GTT) and an equal volume of *E*. *coli* suspended in LB with 10^5^ CFU/ml or *S*. *aureus* suspended in mannitol salt agar with 10^5^ CFU/ml were preincubated at 37°C for 12 h. About 30 μl of these mixtures was plated with eosin methylene blue agar for *E*. *coli* or mannitol salt agar plate for *S*. *aureus*, 6 plates each. Plates were incubated for 24 h at 37°C before colonies in each plate were counted. For the control, the same procedure was followed, but resistant sera were replaced by LB for *E*. *coli* or mannitol salt agar for *S*. *aureus*. The survival of bacteria was expressed as percent survival relative to the number of cells incubated with LB or mannitol salt agar (100% control). Each sample was tested separately three times [7, 25].

For determination of antibacterial activity in the presence of C3, inhibition experiments were carried out with CTC/GTT serum samples that had been pretreated at 56°C for 30 min, with 10 mM EDTA or with rat anti-bovine C3 (25 μl diluted 10^−3^) [25]. EDTA or antiserum was added to the individual serum samples for 30 min prior to the addition of *E*. *coli* and *S*. *aureus*.

### Fluorescence quantitative real-time PCR (RT-PCR)

Total RNA was isolated from heart, liver, spleen, lung, kidney, and mammary tissues from six Chinese Holstein cattle (three healthy samples and three diseased samples with clinical mastitis) using TRIzol reagent (Bioteke, Beijing, China) in accordance with the manufacturer’s instructions. cDNA was synthesized with the transcriptor first-strand cDNA synthesis kit (TaKaRa, Dalian, China). RT-PCR analysis was performed using a 20 μl mixture containing 50 ng of cDNA, 0.4 μM of sense and antisense primers, 6.8 μl of dH_2_O, 10.0 μl of SYBR® Premix Ex Taq™ (2×), and 0.4 μl of ROX Reference Dye (50×) (TaKaRa, Dalian, China). To normalize differences in the amount of total cDNA added to each reaction, the reaction mixture was denatured for 30 s at 95°C and incubated for 40 cycles (denaturing for 5 s at 95°C, annealing for 31 s at 60°C).

The primers used in the experiment based on *C3* (accession NO. NM_001040469.2) were as follows: F: GAGATTCTGGCCGTGAGCTTG, R: GATCGCTCGGATCTCCACTTG. The PCR was monitored by the ABI PRISM 7000HT Fast Real-Time PCR system. The relative quantification of the *C3* gene expression was calculated using the standard curve-based method for relative RT-PCR.

### Statistical analysis

The distribution frequency of SCS is usually skewness [8], so we calculated the SCS (cells/μl)-based parameter SCS using the following equation: SCS = log_2_ (SCS/100) + 3 [26].

Indirect methods of estimation are commonly used. Milk SCS can be employed for selection of mastitis resistance in cattle breeding instead of a direct index of the mastitis phenotype (Interbull: http://www-interbull.slu.se/national_ges_info2/framesida-ges.htm). This indirect index correlates genetically with the occurrence of clinical mastitis (r = 0.7) [26].

TFPGA software was used for genetic analysis, including calculation of both genotypic and allelic frequencies for each SNP, to test for the Hardy–Weinberg equilibrium (χ^2^) in genotype distributions across breeds and to determine values for polymorphism information content (PIC), heterozygosities (H_e_), and effective number of alleles (N_e_) [27]. SHEsis software was employed to conduct haplotype analysis and linkage disequilibrium analysis [28].

Correlation analyses between the three SNPs (single or combined) of the C3 gene and milk traits, the hemolytic activity, and the concentration in Chinese Holstein were analyzed following the general least-square model:

$$Y_{ijkml}=\mu +G_{i}+YS_{j}+H_{k}+P_{l}+FS_{m}+e_{ijkml}$$

where *Y*_*ijkml*_ = the observed value of each milk trait or serum level trait; *μ* = overall mean; *G*_*i*_ = fixed effect of genotype or combined genotype; *YS*_*j*_ = fixed effect of season; *H*_*k*_ = fixed effect of farm; *P*_*l*_ = fixed effect of parity; *FS*_*m*_ *=* random effect of sire; *e*_*ijkml*_ = random error.

The antibacterial activity analyses were performed using SPSS Statistics 17.0 software. The data were subjected to Student’s independent samples *t*-test to determine the differences between the means of the different groups. Values of *P*<0.05 were considered significant. Multiple comparisons were performed by Duncan’s multiple range test [29].

## Results

### SNPs of the *C3* gene

**Fig 2:**
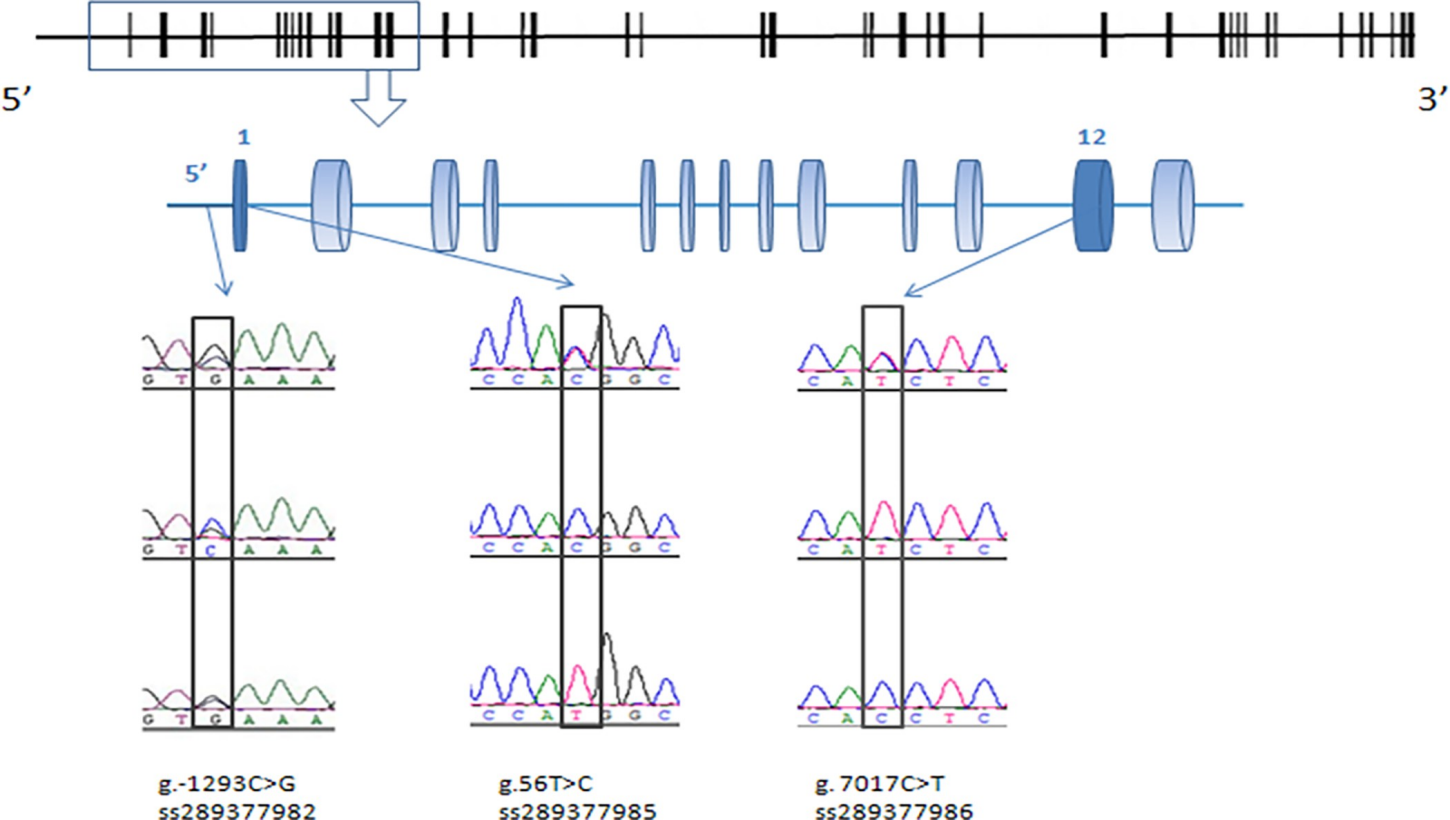
Shows the 5′ region and the exons of the *C3* gene in the vertical line and the three new SNPs that we found by sequencing.

Compared with the sequence of the bovine *C3* gene (GenBank accession NO. NC_007305.4), three new SNPs (g.-1293C>G, g.56T>C, g.7017C>T) were detected in all three tested breeds (Fig 2). One SNP (g.-1293C>G) of the *C3* gene was located in the 5′-flanking regions, and the two other SNPs (g.56T>C, g.7017C>T) in exon I and exon XII, which showed one non-synonymous mutation ATG (Met)>ACG (Thr) (at position 19th aa of signal peptide) and one synonymous mutation CAC (His)>CAT (His) (at position 455th aa), respectively. Three new SNPs were submitted to the National Center for Biotechnology Information (NCBI) database (ss289377982, ss289377985, ss289377986). The SNPs such as C>T(rs43501920), A>G (rs43501919), and C>T(rs43501924) of the bovine C3 gene, which had been deposited on the BLASTN program of the NCBI, were not found in our test cattle. We hypothesized that the differences of the bovine population may cause differences in the SNP loci.

TESS (http://www.cbil.upenn.edu/cgi-bin/tess/tess) and Signal Scan (http://www-bimas.cit.nih.gov/molbio/signal/) were used to confirm the presence of the TATA box and binding sequence motifs by screening the 2 kb 5′-flanking region upstream of the C3 gene. We found that the loci of g.-1293C>G may be involved in the formation of the GC-rich stem-loop structure. This structure participates in the formation of termination mechanism independent of ρ factor of the prokaryotic terminator.

### PCR-RFLP and allele frequencies

**Fig 3:**
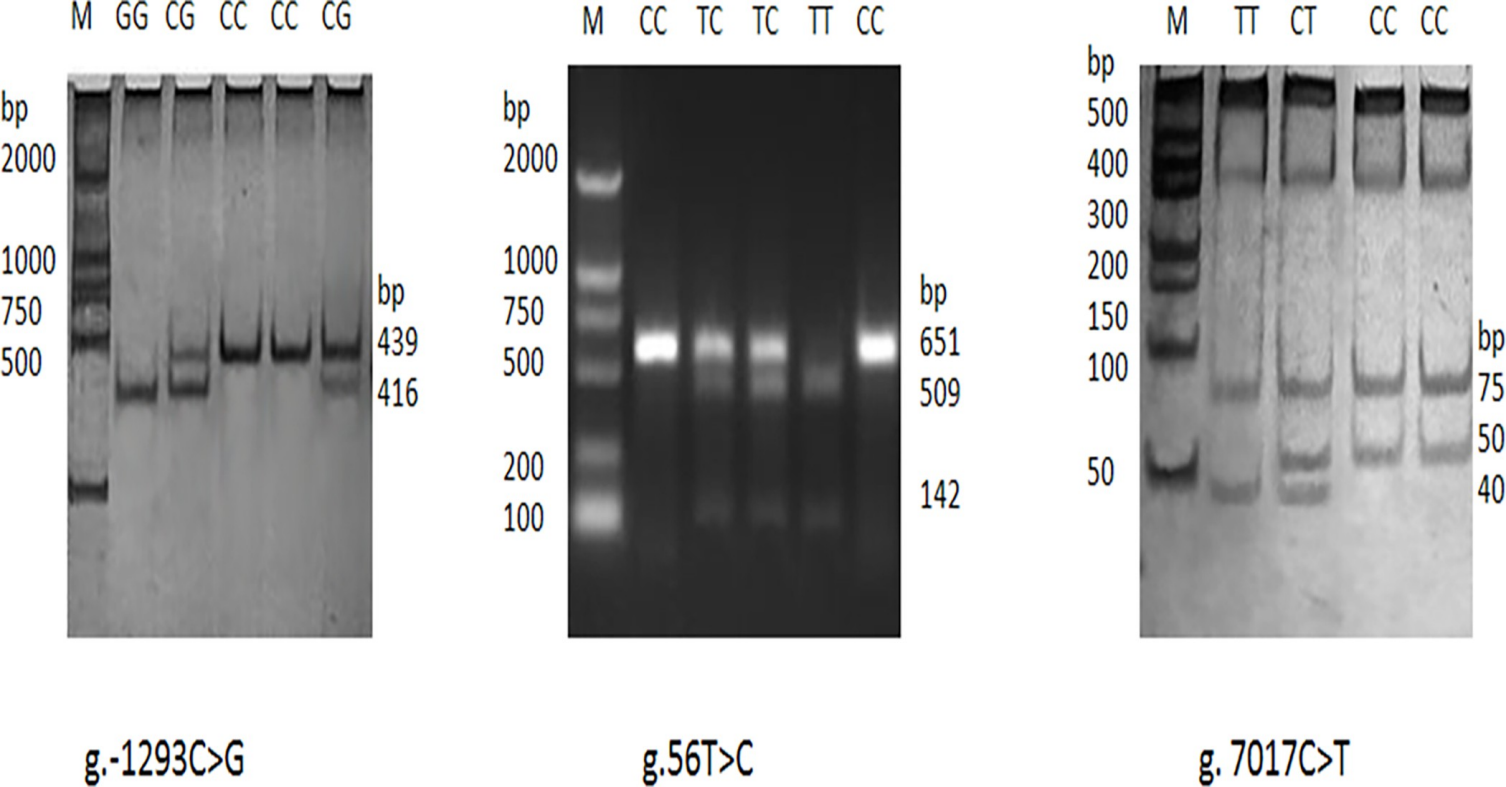
PCR-RFLP detection results of the three new SNPs (g.-1293C>G, g.56T>C, g.7017C>T) in bovine *C3* gene.

The three primers, the respective amplified sizes of fragment, and annealing temperatures for the novel SNPs are shown in Table 2. Digestion of the PCR product by *Hinf* I (containing the C3 g.-1293C>G locus) generated fragments with lengths of 416 and 23 bp for genotype GG; 439, 416, and 23 bp for genotype CG; and 439 bp for genotype CC. Digestion with *Sty* I (containing the *C3* g.56T>C locus) generated fragments of 651 bp for genotype CC; 651, 509, and 142 bp for genotype CT; and 509 and 142 bp for genotype TT owing to a nucleotide substitution. Digestion of the PCR fragment by *Hinf* I (containing C3 g.7017C>T locus) produced two bands (75 and 50 bp), four bands (75, 50, 40, and 10 bp), and three bands (75, 40, and 10 bp) for the CC, CT, and TT genotypes, respectively. The 23 and 10 bp fragments were not noticeable in the gel due to their small size (Fig 3).

The allele and genotype frequencies of the three SNPs in bovine *C3* gene are shown in Table 3. The alleles C, T, and C were the dominant alleles at positions g.-1293C>G, g.56T>C, and g.7017C>T, respectively, in the three breeds. The frequency of alleles C, T, and C varied from 0.76 to 0.8821 at g.-1293C>G, 0.6937 to 0.8585 at g.56T>C, and 0.80 to 0.8289 at g.7017C>T in Chinese Holstein, Luxi Yellow, and Bohai Black breeds, respectively. The homozygote CC genotype at position -1293 was the dominant genotype in Chinese Holstein (0.6991), Luxi Yellow (0.8019), and Bohai Black (0.56) breeds. The heterozygous TC at position 56 was the most common genotype in Chinese Holstein (0.4713), while homozygote TT was the most common genotype in Luxi Yellow (0.717) and Bohai Black (0.64) breeds. At the g.7017C>T locus, the homozygote CC genotype was the dominant genotype in Chinese Holstein (0.6955), Luxi Yellow (0.6132), and Bohai Black (0.60) breeds.

**Table 3:** Genotypic distribution and allelic frequencies of 3 SNPs of the bovine *C3* gene of three breeds.

Loci/ Sample size	Genotypic frequencies			Allelic frequencies	
g. -1293C>G	CC	CG	GG	C	G
CH/821	0.6991	0.2716	0.0292	0.835	0.165
	574	223	24		
LYC/106	0.8019	0.1604	0.0377	0.8821	0.1179
	85	17	4		
BH/25	0.56	0.40	0.04	0.76	0.24
	14	10	1		
g. 56T>C	TT	TC	CC	T	C
CH/821	0.4579	0.4713	0.0708	0.6937	0.3063
	376	387	58		
LYC/106	0.717	0.283	0	0.8585	0.1415
	76	30	0		
BH/25	0.64	0.36	0	0.82	0.18
	16	9	0		
g. 7017C>T	CC	CT	TT	C	T
CH/821	0.6955	0.2667	0.0378	0.8289	0.1711
	571	219	31		
LYC/106	0.6132	0.3774	0.0094	0.8019	0.1981
	65	40	1		
BH/25	0.60	0.40	0	0.80	0.20
	15	10	0		

Note: “CH” means Chinese Holstein breed, “LYC” means Luxi Yellow breed,“BH” means Bohai Black breed.

The genetic index was evaluated in the three breeds. The value of χ^2^ test, H_e_, N_e_, PIC, and Shannon’s information index are presented in Table 4. The result of the χ^2^ test indicated that only position g.-1293C>G did not meet the Hardy–Weinberg equilibrium (*P<0*.*05*) in Luxi Yellow breed, while SNPs g.56T>C and g.7017C>T in all three breeds all met the Hardy–Weinberg equilibrium (*P>0*.*05*).

**Table 4:** χ^2^ tests, heterozygosities (He), effective number of alleles (Ne) and polymorphism information contents (PIC) of *C3* gene of three breeds.

Loci	Breed/Sample	χ^2^	PIC	He	Ne	Hardy-Weinberg Equilibrium
g. -1293C>G	CH/821	0.1718	0.2376	0.2756	1.3805	*P > 0*.*05*
	LYC/106	5.5632	0.1864	0.208	1.2627	*P < 0*.*05*
	BH/25	0.2328	0.2983	0.3648	1.5743	*P > 0*.*05*
g. 56T>C	CH/821	2.1975	0.3289	0.4149	1.7092	*P > 0*.*05*
	LYC/106	2.8801	0.2135	0.243	1.3209	*P > 0*.*05*
	BH/25	1.2046	0.2516	0.2952	1.4188	*P > 0*.*05*
g. 7017C>T	CH/821	2.929	0.2435	0.2837	1.396	*P > 0*.*05*
	LYC/106	3.7335	0.2673	0.3177	1.4657	*P > 0*.*05*
	BH/25	1.5625	0.2688	0.32	1.4706	*P > 0*.*05*

Abbreviation: CH = Chinese Holstein breed; LYC = Luxi Yellow breed; BB = Bohai Black breed; *P>0*.*05* means equilibrium and *P<0*.*05* means disequilibrium.

The linkage disequilibrium estimation indicated that the three SNPs were unlinked in the 821 Chinese Holstein cattle (D’<0.75, r^2^<0.33) (Table 5).

**Table 5:** Analysis of pairwise linkage disequilibrium in *C3* gene.

	g.-1293C>G	g.56T>C	g.7017C>T
g.-1293C>G	-	0.038	0.548
g.56T>C	0	-	0.123
g.7017C>T	0.012	0.001	-

Note: D’ is above the diagonal for SNPs and r^2^ is below the diagonal.

### Relationships between SNPs, combined genotypes of C3 gene, and Chinese Holstein cow milk production traits

Table 6 summarizes the effects on milk production traits (rates of fat and protein and milk yield at 305-days) and SCSs. Polymorphism at g.56T>C was strongly correlated with SCS (*P<0*.*05*). Cattle that showed the homozygous genotype CC, rather than TC or TT, at position 56 also showed significantly lower SCS (*P<0*.*05*). Polymorphism at g.-1293C>G was related to protein rate for studied Chinese Holstein cattle (*P<0*.*05*). The homozygous genotype GG at position -1293 had significantly higher protein content than those with genotypes of CC and CG (*P<0*.*05*). However, no significant correlation between g.-1293C>G/g.7017C>T polymorphism and SCS/milk traits was found in 821 Chinese Holstein cattle (*P>0*.*05*) (Table 6).

**Table 6:** Least squares mean (LSM) and standard errors (SE) for SCS, fat, protein rate and milk yield of different *C3* genotypes in 821 Chinese Holstein.

SNP	Genotype/Samples	Somatic cell score	Protein Content (%)	Fat Content (%)	305 d milk yield (kg)
g.-1293C>G	CC/574	4.12±0.26	3.00±0.06^b^	3.51±0.19	5849.16±316.52
	CG/223	4.12±0.28	2.92±0.06^b^	3.37±0.20	5667.83±340.01
	GG/24	4.21±0.47	3.02±0.11^a^	3.38±0.34	6216.51±573.31
g. 56T>C 791	TT/376	4. 17±0.23^a^	2.94±0.07	3.36±0.20	5892.04±344.77
	TC/387	4. 06±0.24^a^	2.96±0.07	3.39±0.21	5831.76±356.72
	CC/58	3.65±0.33^b^	3.03±0.09	3.31±0.27	6009.70±454.95
g.7017C/T	CC/571	4.06±0.23	2.97±0.06	3.30±0.19	5965.12±329.57
	CT/219	4.01±0.25	2.90±0.07	3.32±0.21	5938.00±360.40
	TT/31	4.42±0.38	3.07±0.10	3.62±0.31	5830.40±513.74

Note: Mean values with the different small letters within the same row and loci differ at *P*<0.05.

The polymorphism at g.56T>C and g.7017C>T was significantly correlated with serum ACH50 values in 354 Chinese Holstein cows (*P<0*.*01*) (Table 7). The homozygous genotype CC at position 56 had significantly higher ACH50 than the genotypes of TC and TT (*P<0*.*01*), while the homozygous genotype CC at position 7017 had significantly lower ACH50 than genotypes of CT and TT (*P<0*.*01*). The correlation between g.-1293C>G polymorphism and ACH50 was insignificant for the 354 Chinese Holstein cattle (*P>0*.*05*).

**Table 7:** Least squares mean (LSM) ± standard errors (SE) of various milk production traits in relation to different C3 genotypes among 354 Chinese Holstein cows.

SNP Locus	Genotype /samples	C3 level (mg/ml)	CH50 (U/ml)	ACH50 (U/ml)
g.-1293C>G	CC/263	2.50±0.67	68.46±4.11	29.50±1.25
	CG/86	2.98±0.73	64.61±4.51	28.34±1.37
	GG/5	2.84±1.92	62.35±11.84	28.94±3.60
g. 56T>C	TT/184	2.77±0.66	67.65±4.09	29.42±1.2^B^
	TC/151	2.38±0.72	66.40±4.45	28.33±1.34^B^
	CC/19	2.44±1.14	64.60±7.03	32.98±2.11^A^
g.7017C>T	CC/236	2.45±0.66	68.63±4.07	28.25±1.23^B^
	CT/95	3.23±0.74	62.84±4.53	31.25±1.37^A^
	TT/23	2.10±1.06	73.60±6.52	29.82±1.96^A^

Note: Mean values superscripted with “A” are very significantly higher than those superscripted with “B”, *P<0*.*01* within the same row and loci.

The C3 SNPs (g.-1293C>G, g.56T>C, and g.7017C>T) were used for haplotype reconstruction, including H1: CCC, H2: CCT, H3: CTC, H4: CTT, H5: GCC, H6: GCT, H7: GTC, and H8: GTT. The estimated frequencies were 0.208, 0.040, 0.476, 0.113, 0.043, 0.002, 0.108, and 0.009 for H1–H8, respectively. Among them, H6 was the least common, while H3 was the most common. Although 27 combined haplotypes were possible across the three SNPs, only 21 combinations were observed in the studied Chinese Holstein individuals.

Six combined genotypes, H1H6(3), H2H2(2), H2H8(2), H4H8(3), H5H8(1), and H7H8 (2), were not included in the association analysis due to the small sample sizes (<4). Statistical results showed that the fat rate, SCS, and protein rate in combinations of haplotype (three SNPs) are significantly different (*P<0*.*05*) in the 821 tested Chinese Holstein population (Table 7). No significant difference in 305-day milk yield was determined in various haplotype combinations (Table 8). The individuals with haplotype combination of H1H5 presented a lower SCS than those with haplotype combinations of H1H8 and H4H4 (*P<0*.*05*). The individuals with haplotype combinations of H1H1 and H4H4 showed significantly higher protein rate than those with haplotype combinations of H1H4, H1H8, H3H4, and H3H7 (*P<0*.*05*). The individuals with haplotype combination of H1H1 had significantly higher fat rate than those with haplotype combination of H1H8 (*P<0*.*05*).

**Table 8:** Least squares mean (LSM) and standard error (SE) for SCC、fat、protein and milk yield of different *C3* haplotype combinations in 821 Chinese Holstein.

Haplotype combination/Samples	Somatic cell score	Protein Content (%)	Fat Content (%)	305 d milk yield (kg)
H1H1/27	3.96±0.40	3.10±0.09^a^	3.45±0.29^a^	6039.00±501.34
H1H2/15	4.18±0.63	3.02±0.14	3.43±0.44	5288.94±733.87
H1H3/171	4.06±0.26	2.97±0.06	3.36±0.19	5803.80±322.12
H1H4/62	3.96±0.31	2.89±0.07^b^	3.40±0.23	5711.16±381.00
H1H5/12	2.49±0.54^b^	2.85±0.13	2.86±0.39	5866.80±669.71
H1H7/89	4.23±0.29	2.93±0.07	3.32±0.21	5514.39±363.30
H1H8/18	4.63±0.45^a^	2.83±0.11^b^	2.71±0.32^b^	5643.32±554.15
H2H4/7	4.49±0.64	2.88±0.15	3.43±0.46	6597.67±787.14
H3H3/187	3.97±0.25	2.95±0.06	3.13±0.18	5911.60±315.66
H3H4/84	4.02±0.30	2.88±0.07^b^	3.26±0.22	5839.48±376.34
H3H7/83	3.97±0.30	2.83±0.07^b^	3.19±0.22	5636.13±372.92
H3H8/20	3.35±0.51	2.92±0.12	2.73±0.37	5828.42±614.93
H4H4/17	4.52±0.49^a^	3.18±0.12^a^	3.49±0.36	5076.86±607.42
H5H7/7	3.79±0.67	2.87±0.16	3.20±0.49	6310.23±825.53
H7H7/11	3.86±0.59	3.04±0.14	2.86±0.43	5847.63±727.69

Abbreviation: H1 = CCC, H2 = CCT, H3 = CTC, H4 = CTT, H5 = GCC, H6 = GCT, H7 = GTC and H8 = GTT. Means with the different small letters within the same row differ at *P* < 0.05 and means without any superscript do not differ statistically (*P* > 0.05).

No significant difference in C3 concentration and CH50 305-day milk yield was observed in various haplotype combinations in 354 Chinese Holstein cows (Table 9). However, the individuals with haplotype combination of H1H2 showed higher ACH50 value than those with haplotype combinations of H1H3 (*P = 0*.*0008*) and H3H7 (*P = 0*.*001*) in 354 Chinese Holstein cows.

**Table 9:** Least squares mean (LSM) + standard error (SE) of C3 concentration, ACH50 and CH50 values in different haplotypic combinations among 354 Chinese Holstein cows.

Haplotype combination /Numbers	C3 Concentration (μg/ml)	CH50 (U/ml)	ACH50 (U/ml)
H1H1/11	2.75±1.39	71.09±8.57	29.64±2.54
H1H2/6	1.92±1.79	58.07±11.01	41.44±3.26^A^
H1H3/68	1.66±0.82	69.17±4.99	27.08±1.48^B^
H1H4/33	3.49±0.99	64.74±6.05	29.54±1.79
H1H7/32	2.36±0.98	65.44±6.02	28.52±1.78
H1H8/5	1.53±1.92	41.47±11.75	30.68±3.48
H2H4/7	1.72±1.71	76.83±10.51	31.18±3.12
H3H3/82	2.17±0.78	70.19±4.79	29.16±1.42
H3H4/44	3.14±0.87	66.08±5.34	31.39±1.58
H3H7/37	3.50±0.81	68.34±5.59	27.26±1.65^B^
H3H8/6	2.88±1.77	54.78±10.84	29.91±3.21
H4H4/12	1.60±1.38	71.67±8.46	28.82±2.51
H5H7/4	2.91±2.14	67.70±13.12	29.19±3.89

Abbreviations: H1 = CCC, H2 = CCT, H3 = CTC, H4 = CTT, H5 = GCC, H6 = GCT, H7 = GTC and H8 = GTT. Note: Mean values superscripted with “A” are very significantly higher than those superscripted with “B”, *P<0*.*01* within the same row and loci.

### C3 gene expression

**Fig 4:**
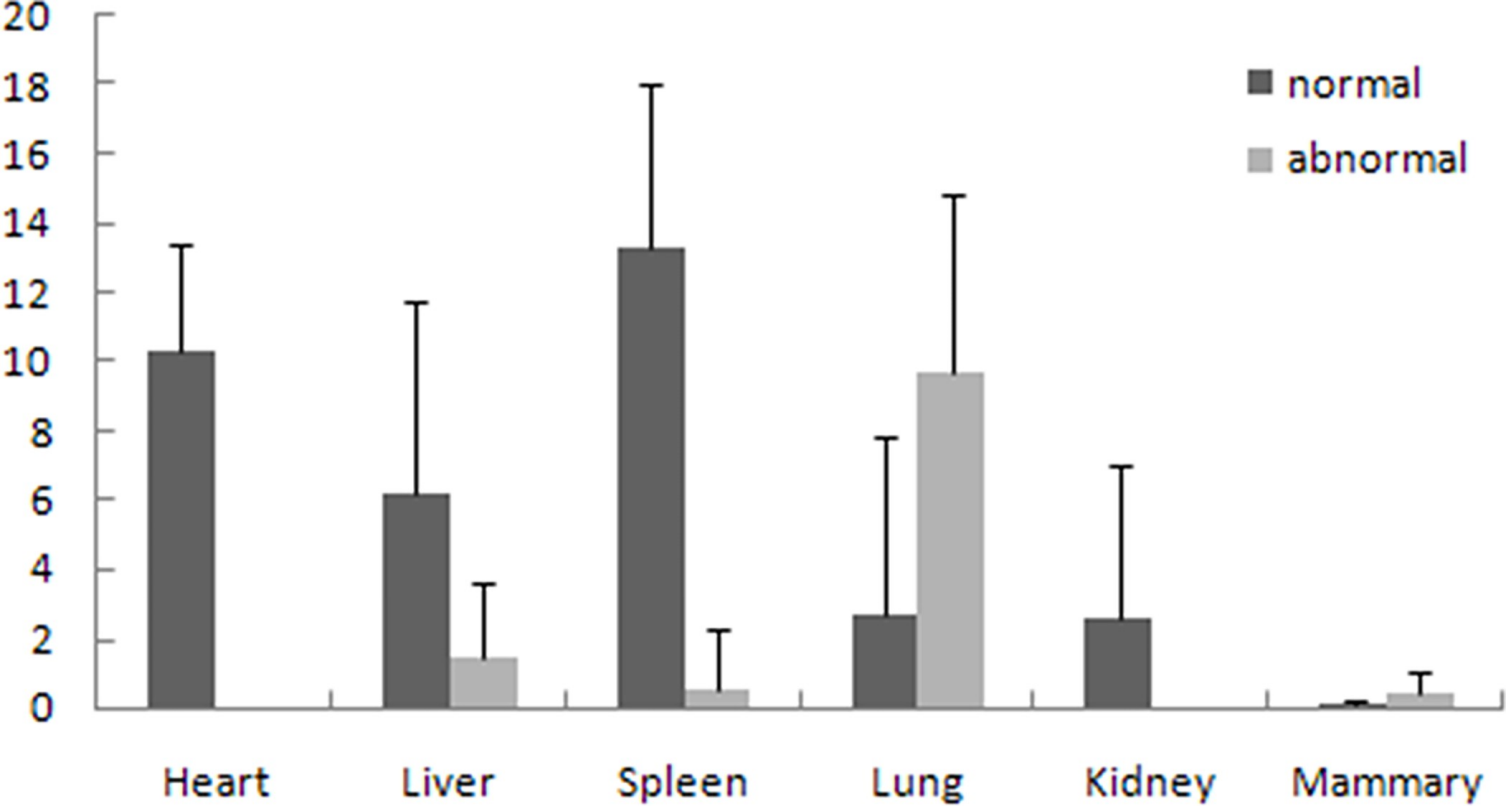
C3 expression in different tissues of healthy cow samples and diseased samples.

The C3 gene expression levels differed across tissues between healthy and mastitic cattle (Fig 4). Quantitative data indicate that the spleen, heart, and liver tissues had higher *C3* expression than the mammary tissues in healthy Chinese Holstein cows (normal group). However, the C3 expression levels were significantly increased in lung and mammary tissues in mastitic cows (abnormal group) compared with those in healthy animals (*P*<0.05), while the C3 expression levels were significantly decreased in heart, spleen, liver, and kidney tissues in mastitic cows (abnormal group) compared with those in healthy animals (*P*<0.01).

### C3 antibacterial activity

**Fig 5:**
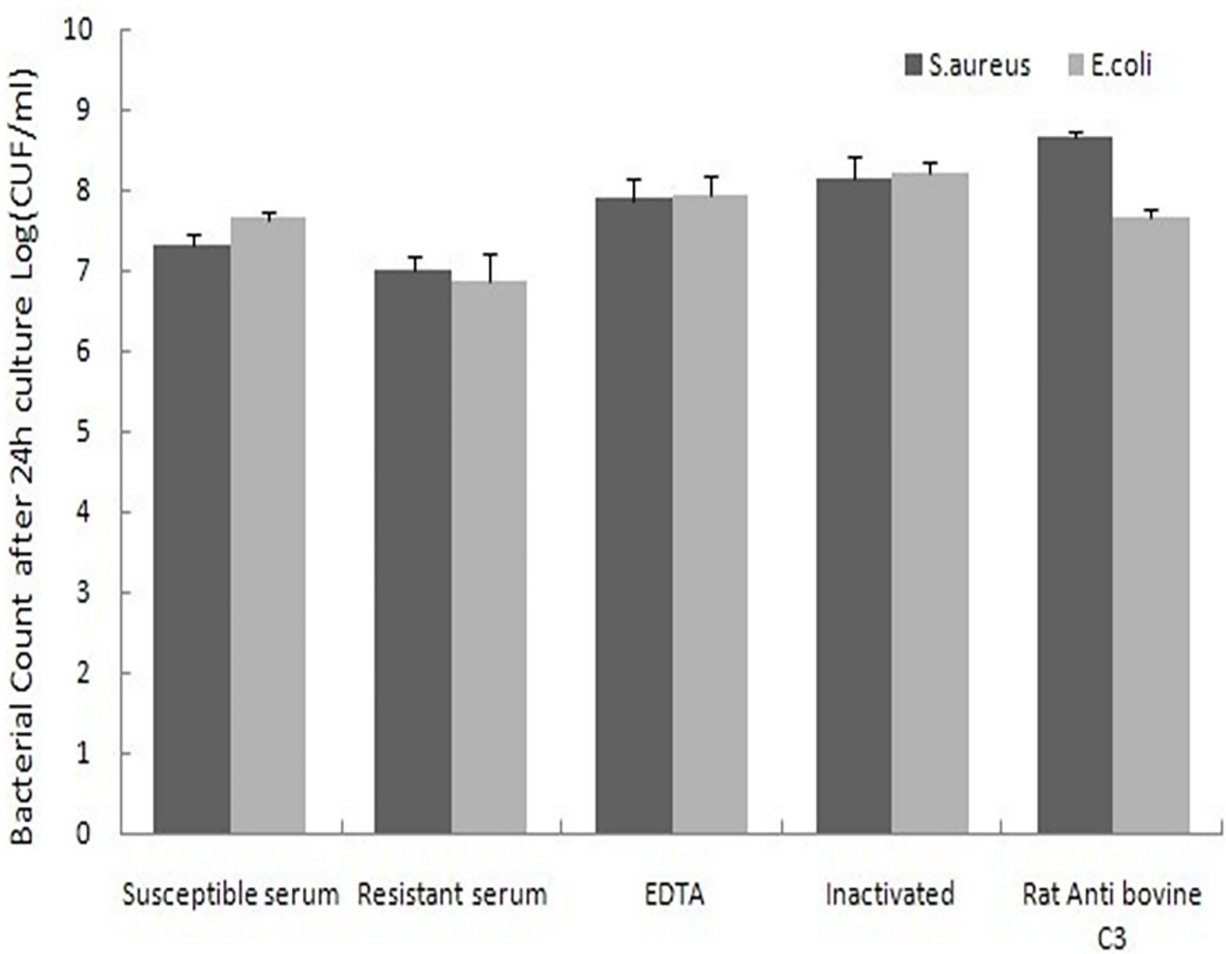
Serum antibacterial activity of resistant and susceptible cows and neutralization of antibacterial activity from resistant subjects by specific inhibition of C3. The differences in Log (CFU/ml) between resistant serum and others were highly significant (*P*<0.001). Data are means ± standard deviation.

Genetically resistant cows (H1H5) had serum with noticeably higher antibacterial activity against *S*. *aureus* and *E*. *coli* (Fig 4) in vitro than genetically susceptible H1H8 cows (*P*<0.05). Resistant serum (H1H5) that was preincubated with EDTA, heated at 56°C for 30 min, or treated with C3 antibody lost antibacterial activity against both tested strains (Fig 5).

## Discussion

Complement, one of the essential effective systems of innate immunity, is present in lower animals such as fish and higher animals such as humans and bovines [30]. The complement system plays a crucial role in innate defense against pathogens and serves as a functional bridge with adaptive immunity to allow integrated host defense [31]. It is a potential therapeutic target for improved general immune response in many species. In the current study, we scanned most exons of the *C3* sequence to study the associations between polymorphisms in the C3 gene with the productive traits and immune response traits [18].

The SNP (g.56T>C) in exon I showed one non-synonymous mutation ATG (Met)>ACG (Thr). The loci are at position 19th aa of the signal peptide, which is the C-terminal near the side of action of the signal peptidase. The C-terminal plays an essential role in combining the C3 protein with signal recognition particle, indicating that the region affects the C3 secretion [32]. Twenty-one primer pairs were designed based on DNA sequence of the bovine *C3* gene (GenBank accession NO. NC_007305.4) and synthesized to find the SNPs (Table 1). More than 18,000 bp had been detected, only three SNPs have been found in 952 individuals of Chinese Holstein, Luxi Yellow, and Bohai Black breeds. Thus, the gene may be relatively conservative. However, the three breeds did not show all three different genotypes: the Luxi Yellow and Bohai Black cattle with the SNP (g.56T>C) only showed the TT and TC genotypes, while Bohai Black cattle with the SNP (g.7017C>T) only showed the CC and CT genotypes (Table 3). The χ^2^ test results showed that the three SNPs all accord with the Hardy–Weinberg disequilibrium (*P*>0.05), except for the Luxi Yellow cattle with the SNP (g.-1293C>G) (Table 4). This finding implies that the three SNPs become stable after long-term artificial selection in the chosen samples.

The SNP (g.-1293C>G) is significantly associated with the protein content (Table 6), and the protein content of cows with g.-1293C>G-GG genotype is considerably higher than that of cows with g.-1293C>G-CC and -CG genotypes (*P*<0.05). The SCS of cows with g.56T>C-CC genotype was significantly lower than that of cows with g.56T>C-TC and -TT genotypes (*P*<0.05). However, the ACH50 value of cows with g.56T>C-CC genotype was significantly higher than that of cows with g.56T>C-TC and -TT genotypes (*P*<0.01). The allelic frequencies of G and C of g.-1293C>G and g.56T>C are 16.5% and 29.38%, respectively (Table 3). The results confirmed the importance of the two SNPs, which are better for animals to resist pathogens.

The study wanted to determine why the value of ACH50 was significantly associated with cows with the SNP (g.7017C>T) (*P<0*.*01*) (Table 3) and cows with CC have significantly lower ACH50 than those with TT and CT. A previous study [33] found that rare silent mutation can affect protein function in some cellular environments. It may be due to the T allele leading to some three-dimensional structure change of the C3 beta chain and involving the process or the other protein to combine it.

In this study, individuals with the haplotype combination of H1H1 (CCC/CCC) showed higher protein and fat contents (*P<0*.*05*) than those with haplotype combinations of H1H8 (CCC/GTT) (*P<0*.*05*). Given that individuals with haplotype combination of H1H5 (CCC/GCC) showed significantly lower SCSs and the lowest C3 concentration in serum than individuals with haplotype combinations of H1H8 (CCC/GTT) (*P = 0*.*0007*) and H4H4 (CTT/CTT) (*P* = 0.0021), we can conclude that H1H8 (CCC/GTT) should be abandoned. Cattle with H1H1 (CCC/CCC) and H1H5 (CCC/GCC) haplotype combinations can be selected for breeding to obtain low SCS. Cattle with H1H1 (CCC/CCC) and H1H5 (CCC/GCC) haplotype combinations can be selected for breeding to obtain low SCS. Cows with haplotype combination of H2H4 (CCT/CTT) had a higher value of CH50 and 305-day milk yield than other cows (*P>0*.*05*). The individuals with haplotype combination of H1H2 (CCC/CCT) had significantly higher ACH50 than those with haplotype combinations of H1H3 (CCC/CTC) (*P = 0*.*0008*) and H3H7 (CTC/GTC) (*P = 0*.*001*). Thus, cows having haplotype combinations of H2H4 and H1H2 can be used for breeding to become more resistant to pathogens. A previous study [34] reported the crystal structure of the C3 convertase formed by C3b and the protease fragment Bb, which was stabilized by the bacterial immune-evasion protein SCIN (Staphylococcal complement inhibitor). Thus, the SCIN from *S*. *aureus* can obstruct the activation of C3 protein and can affect a series of reactions caused by C3 activation [34]. The results of the preliminary test of antibacterial activity of C3 (Fig 5) further verified that cows with the haplotype combination of H1H5 (CCC/GCC) are more resistant than those with H1H8 (CCC/GTT). The bacterial count increased when resistant serum samples with the H1H5 (CCC/GCC) haplotype combination were incubated with EDTA and C3 antibody samples and treated at 56°C for 30 min (Fig 5). This finding indicates that C3 protein plays an indispensable role in the complement system.

Mastitis resistance is a complex trait. It depends on a genetic component and physiological and environmental factors, including infectious pressure Intramammary infections with *E*. *coli* often result in acute mastitis with severe clinical consequences [3]. Some *S*. *aureus* infections may also cause subclinical mastitis with no apparent clinical symptoms (e.g., swelling of the udder, milk flakes, fever) and only intermittent detection of bacteria in milk paired with elevated somatic cell counts. Our findings demonstrate an association between the C3 gene and hemolytic complement activity. Although complement is a complex multigene process, which serves in the defence against many pathogenic agents, this aspect of complement is now better understood, reinforcing the overall importance of C3 as a candidate gene for natural forms of defence against various microorganisms.

For humans, homozygous deficiency at the C3 locus is a rare autosomal disease characterized by increased susceptibility to bacteria such as *Neisseria meningitidis*, *Streptococcus pneumoniae*, and *Haemophilus influenzae* [35, 36] or viral infections [37]. C3 plays a vital role as an opsonin for bacteria during the early years of life and is less critical in adults, in whom protective antibodies and anamnestic responses have developed [38]. Several studies have investigated the association of polymorphisms in the C3 component of the male complement system and the susceptibility of some of its alleles to some diseases. The Human serum complement C3 component is the core of the complement system. Several receptor sites of the C3 molecule act broadly with pathogens, blood cells, and stromal cells [39, 40], especially the sites that bind to the type I receptor of the erythrocyte complement (CR1, CD35) and the complement type III receptor of the monocytes and macrophages (CR3, CD11b/CD18) [36]. Thus, C3 mediates the adhesion of pathogens, killing and clearing the immune complex in vivo as a bridge [37].

The study indicates that the C3 gene plays a role in resistance to bacterial infection and can be used as a molecular marker for complement activity and traits related to milk production. This study is the first to describe the association between polymorphisms in the bovine C3 gene and milk production traits and complement activity, which has never been done before. Our study investigated the bovine C3 as a candidate gene for complement activity and provides valuable information for further research. Therefore, the compound effect of haplotypes and combined genotypes should be analyzed instead of single locus.

## Supporting information

S1 Fig Bohai Black cattle. (TIFF)

S2 Fig Chinese Holstein cattle. (TIFF)

S3 Fig Luxi Yellow cattle. (TIFF)

S1 Raw images (PDF)
