# Supplementary figures and images for: Complement component 3 haplotypes influence serum complement activity and milk production traits in Chinese Holstein cattle
Source: PLoS One. 2022 Jun 30;17(6):e0268959. doi: 10.1371/journal.pone.0268959 (PMC9246146; doi:10.1371/journal.pone.0268959)

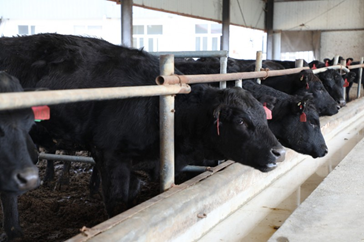

Supplement: S1 Fig — (TIFF) [file pone.0268959.s001.tiff]

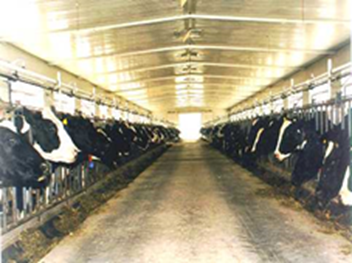

Supplement: S2 Fig — (TIFF) [file pone.0268959.s002.tiff]

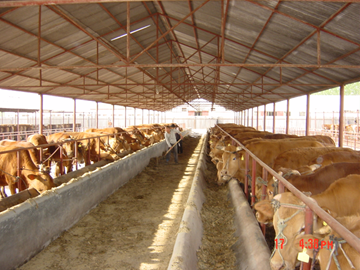

Supplement: S3 Fig — (TIFF) [file pone.0268959.s003.tiff]
