# Supplementary material for: Complement component 3 haplotypes influence serum complement activity and milk production traits in Chinese Holstein cattle
Source: PLoS One. 2022 Jun 30;17(6):e0268959. doi: 10.1371/journal.pone.0268959 (PMC9246146; doi:10.1371/journal.pone.0268959)
Supplement: S1 Raw images — (PDF) [file pone.0268959.s004.pdf]

## Figures

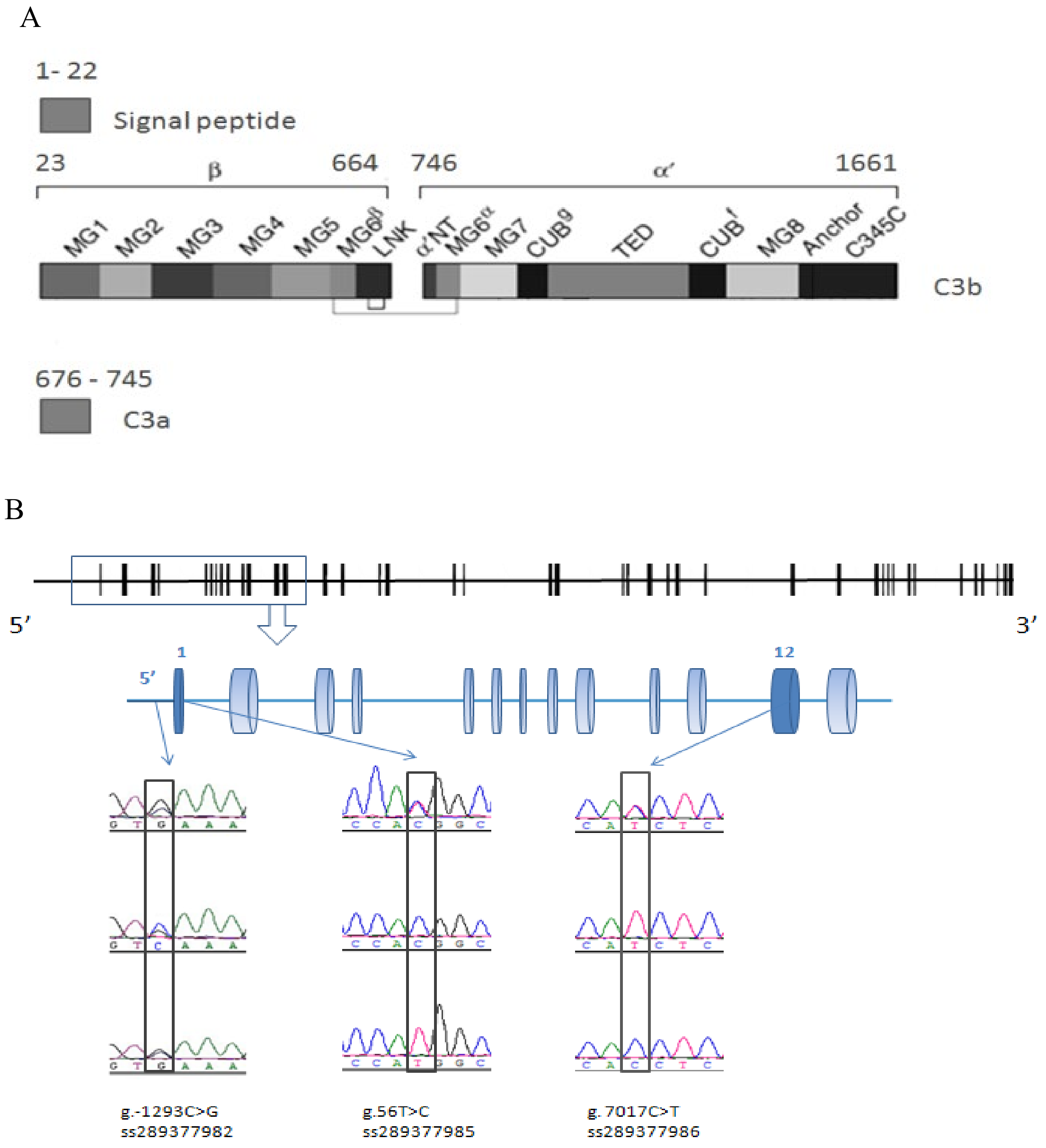


**Fig. 1** A shows the protein fragment of C3: the signal peptide and the alpha and beta chains, which consist of the C3a and C3b fragment. B shows the 5′ region and the exons of the *C3* gene in the vertical line and the three new SNPs that we found by sequencing.

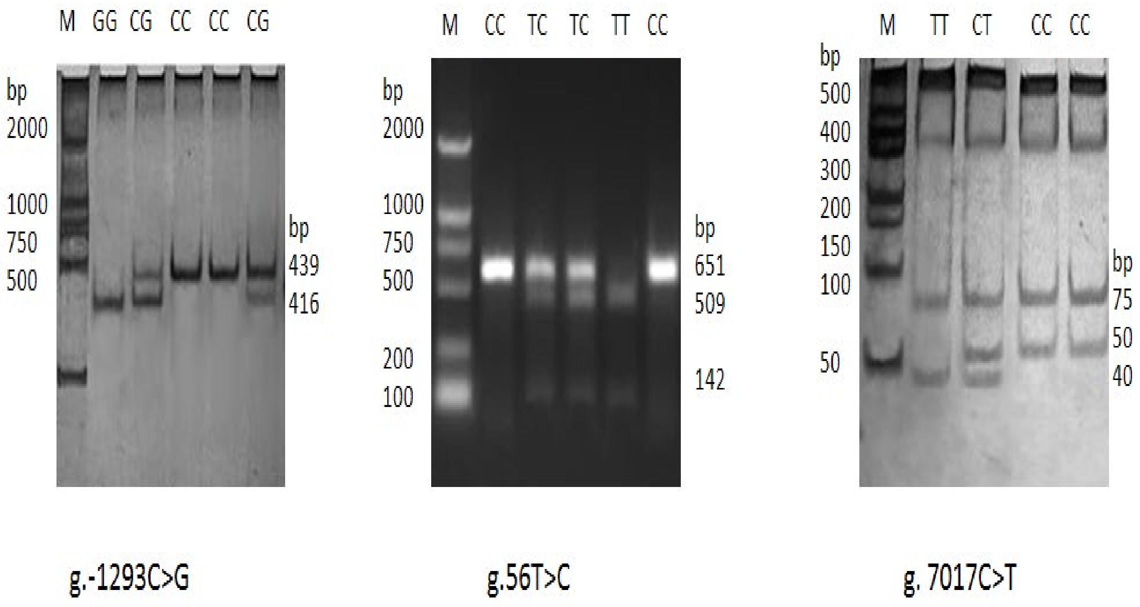


**Fig. 2** PCR-RFLP detection results of the three new SNPs (g.-1293C>G, g.56T>C, g.7017C>T) in bovine *C3* gene.

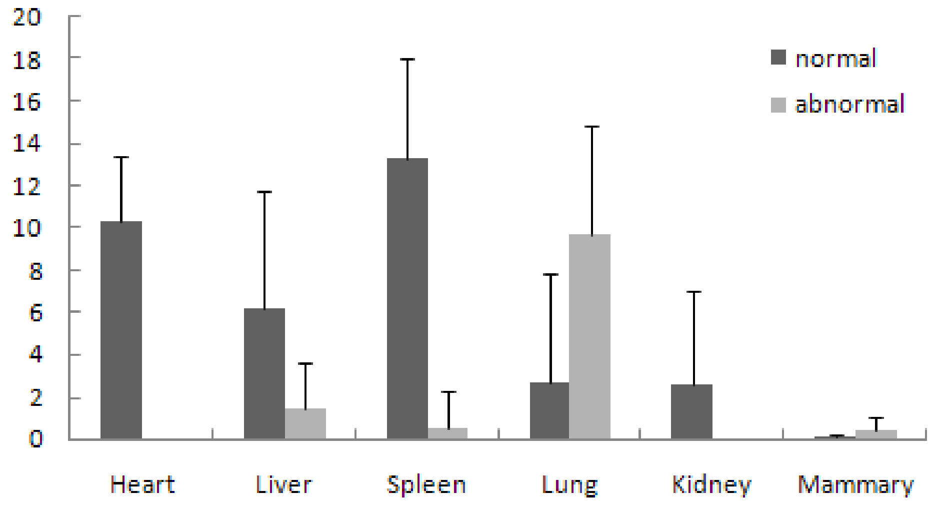


**Fig. 3** C3 expression in different tissues of healthy cow samples and diseased samples. Data are means ± standard deviation.

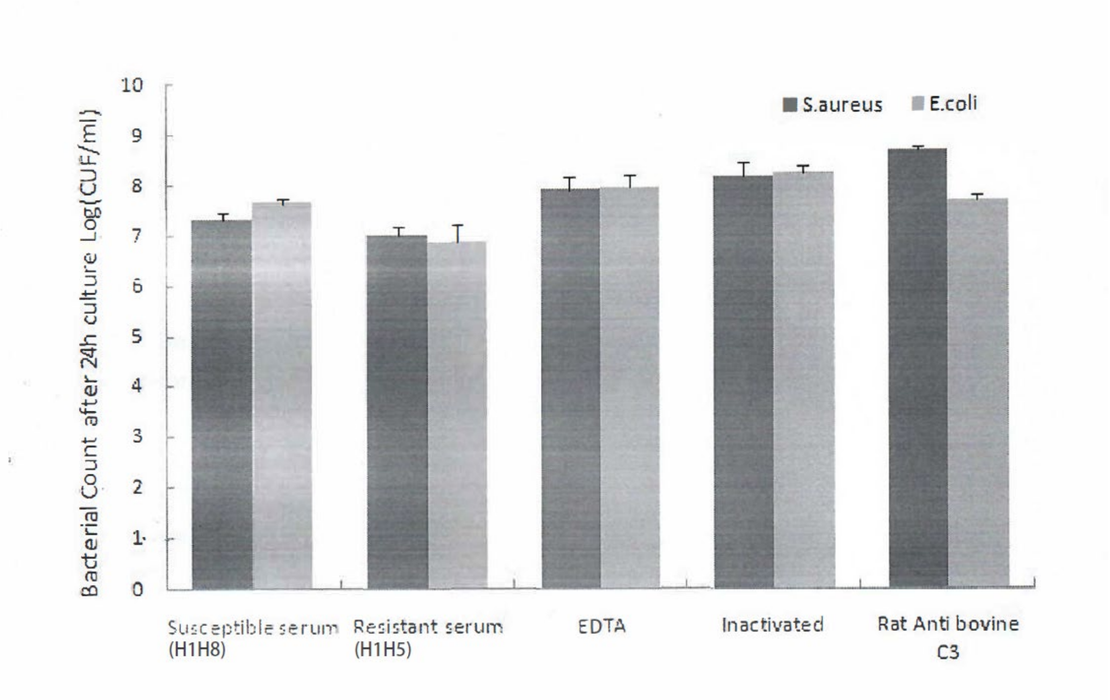


**Fig. 4** Serum antibacterial activity of resistant and susceptible cows and neutralization of antibacterial activity from resistant subjects by specific inhibition of C3. The differences in Log (CFU/ml) between resistant serum and others were highly significant ($P<0.001$). Data are means ± standard deviation.
