# Supplementary material for: Complement component 3 haplotypes influence serum complement activity and milk production traits in Chinese Holstein cattle
Source: PLoS One. 2022 Jun 30;17(6):e0268959. doi: 10.1371/journal.pone.0268959 (PMC9246146; doi:10.1371/journal.pone.0268959)
Supplement: Attachment — Submitted filename: 3.2_Reponse Letter.docx [file pone.0268959.s005.docx]

Dear editor,

We appreciate your comments. The followings are our point-by-point responses:

1. In your cover letter, please note whether your blot/gel image data are in Supporting Information or posted at a public data repository, provide the repository URL if relevant, and provide specific details as to which raw blot/gel images, if any, are not available. Email us at plosone@plos.org if you have any questions.

**A: Thanks for the suggestion. We have already added them in cover letter.**

2. Thank you for updating your data availability statement. You note that your data are available within the Supporting Information files, but no such files have been included with your submission. At this time, we ask that you please upload your minimal data set as a Supporting Information file, or to a public repository such as Figshare or Dryad. Please also ensure that when you upload your file you include separate captions for your supplementary files at the end of your manuscript.

As soon as you confirm the location of the data underlying your findings, we will be able to proceed with the review of your submission.

**A:** **Thanks for the suggestion. We have already added data availability statement in the manuscript and uploaded our data in Supporting Information file.**

**Acknowledgement**

Our deepest gratitude goes to the editors for their careful work and suggestions that improved the manuscript substantially.

Changfa Wang

PhD, Professor

Liaocheng Research Institute of Donkey High-Efficiency Breeding and Ecological Feeding,

Liaocheng University, Liaocheng city, Shandong Province, 252000, P.R. China.
