# Supplementary material for: Complement component 3 haplotypes influence serum complement activity and milk production traits in Chinese Holstein cattle
Source: PLoS One. 2022 Jun 30;17(6):e0268959. doi: 10.1371/journal.pone.0268959 (PMC9246146; doi:10.1371/journal.pone.0268959)
Supplement: Attachment — Submitted filename: 4.17_Reponse Letter.docx [file pone.0268959.s006.docx]

Dear editor,

We appreciate your comments. The followings are our point-by-point responses:

Q: Please include your tables as part of your main manuscript and remove the individual files. Please note that supplementary tables (should remain/ be uploaded) as separate "Supporting Information" files.

**A: Thanks for the suggestion. We have already added table in main manuscript and removed the individual files from “Supporting Information” part.**

**Acknowledgement**

Our deepest gratitude goes to the editors for their careful work and suggestions that improved the manuscript substantially.

Changfa Wang

PhD, Professor

Liaocheng Research Institute of Donkey High-Efficiency Breeding and Ecological Feeding,

Liaocheng University, Liaocheng city, Shandong Province, 252000, P.R. China.
