# Supplementary material for: Complement component 3 haplotypes influence serum complement activity and milk production traits in Chinese Holstein cattle
Source: PLoS One. 2022 Jun 30;17(6):e0268959. doi: 10.1371/journal.pone.0268959 (PMC9246146; doi:10.1371/journal.pone.0268959)
Supplement: Peer review history

# Peer review: Decision Letter 0

**DOI:** 10.1371/journal.pone.0268959.r001

**Authors:** Tomoyoshi Komiyama

6 Oct 2021

PONE-D-21-26730Complement component 3 haplotypes influence serum complement activity and milk production traits in Chinese Holstein cattlePLOS ONE

Dear Dr. Wang,

Thank you for submitting your manuscript to PLOS ONE. After careful consideration, we feel that it has merit but does not fully meet PLOS ONE’s publication criteria as it currently stands. Therefore, we invite you to submit a revised version of the manuscript that addresses the points raised during the review process.

Please submit your revised manuscript by Nov 20 2021 11:59PM. If you will need more time than this to complete your revisions, please reply to this message or contact the journal office at plosone@plos.org. When you're ready to submit your revision, log on to https://www.editorialmanager.com/pone/ and select the 'Submissions Needing Revision' folder to locate your manuscript file.

Please include the following items when submitting your revised manuscript:

- A rebuttal letter that responds to each point raised by the academic editor and reviewer(s). You should upload this letter as a separate file labeled 'Response to Reviewers'.
- A marked-up copy of your manuscript that highlights changes made to the original version. You should upload this as a separate file labeled 'Revised Manuscript with Track Changes'.
- An unmarked version of your revised paper without tracked changes. You should upload this as a separate file labeled 'Manuscript'.

If you would like to make changes to your financial disclosure, please include your updated statement in your cover letter. Guidelines for resubmitting your figure files are available below the reviewer comments at the end of this letter.

If applicable, we recommend that you deposit your laboratory protocols in protocols.io to enhance the reproducibility of your results. Protocols.io assigns your protocol its own identifier (DOI) so that it can be cited independently in the future. For instructions see: https://journals.plos.org/plosone/s/submission-guidelines#loc-laboratory-protocols. Additionally, PLOS ONE offers an option for publishing peer-reviewed Lab Protocol articles, which describe protocols hosted on protocols.io. Read more information on sharing protocols at https://plos.org/protocols?utm_medium=editorial-email&utm_source=authorletters&utm_campaign=protocols.

We look forward to receiving your revised manuscript.

Kind regards,

Tomoyoshi Komiyama, Ph.D

Academic Editor

PLOS ONE

Journal Requirements:

When submitting your revision, we need you to address these additional requirements.

1. Please ensure that your manuscript meets PLOS ONE's style requirements, including those for file naming. The PLOS ONE style templates can be found at 

https://journals.plos.org/plosone/s/file?id=wjVg/PLOSOne_formatting_sample_main_body.pdf and 

https://journals.plos.org/plosone/s/file?id=ba62/PLOSOne_formatting_sample_title_authors_affiliations.pdf

2. PLOS ONE now requires that authors provide the original uncropped and unadjusted images underlying all blot or gel results reported in a submission’s figures or Supporting Information files. This policy and the journal’s other requirements for blot/gel reporting and figure preparation are described in detail at https://journals.plos.org/plosone/s/figures#loc-blot-and-gel-reporting-requirements and https://journals.plos.org/plosone/s/figures#loc-preparing-figures-from-image-files. When you submit your revised manuscript, please ensure that your figures adhere fully to these guidelines and provide the original underlying images for all blot or gel data reported in your submission. See the following link for instructions on providing the original image data: https://journals.plos.org/plosone/s/figures#loc-original-images-for-blots-and-gels. 

  

In your cover letter, please note whether your blot/gel image data are in Supporting Information or posted at a public data repository, provide the repository URL if relevant, and provide specific details as to which raw blot/gel images, if any, are not available. Email us at plosone@plos.org if you have any questions.

3. Your ethics statement should only appear in the Methods section of your manuscript. If your ethics statement is written in any section besides the Methods, please move it to the Methods section and delete it from any other section. Please ensure that your ethics statement is included in your manuscript, as the ethics statement entered into the online submission form will not be published alongside your manuscript. 

4. Thank you for submitting the above manuscript to PLOS ONE. During our internal evaluation of the manuscript, we found significant text overlap between your submission and the following previously published works.

- https://linkinghub.elsevier.com/retrieve/pii/S0163445306002076

- http://www.funpecrp.com.br/gmr/year2011/vol10-1/pdf/gmr1038.pdf

- https://pubmed.ncbi.nlm.nih.gov/16831446/

- https://pubs.acs.org/doi/abs/10.1021/acs.jmedchem.7b00882

- https://hal.archives-ouvertes.fr/hal-00902900/file/hal-00902900.pdf

- https://pubs.acs.org/doi/abs/10.1021/pr100336e

We would like to make you aware that copying extracts from previous publications, especially outside the methods section, word-for-word is unacceptable, even for works which you authored. In addition, the reproduction of text from published reports has implications for the copyright that may apply to the publications.

Please revise the manuscript to rephrase the duplicated text, cite your sources, and provide details as to how the current manuscript advances on previous work. Please note that further consideration is dependent on the submission of a manuscript that addresses these concerns about the overlap in text with published work.

We will carefully review your manuscript upon resubmission, so please ensure that your revision is thorough.

Additional Editor Comments:

Dear Authors,

The focus of your study was to investigate the SNPs of the C3 gene and assess their possible associations between SNPs, combined genotypes with SCS, serum C3 concentration, and complement hemolytic activity in 952 cattle from three Chinese Holstein cattle species.

I have some concerns about the manuscript that you might consider. Three reviews below are from reviewers who have pointed out questions for your manuscript.

All reviewers are critical to your manuscript.　

In particular, reviewer 2 and 3 is critical to your manuscript.　

However, I have carefully considered your manuscript up until now.

I decided to major revision based on the three reviewer’s comments.

I think these comments will be very helpful in the revision of your manuscript.

It is unclear what this study reveals and what your results provide and what will be helpful for research in the future.

If these results become clear, I think your research is important for the future of any researchers who aim to better understand your research.

Also, there are some comments from your manuscript.

Please use this for reference as well.

Tomoyoshi Komiyama, Ph.D

[Note: HTML markup is below. Please do not edit.]

Reviewers' comments:

Reviewer's Responses to Questions

**Comments to the Author**

1. Is the manuscript technically sound, and do the data support the conclusions?

The manuscript must describe a technically sound piece of scientific research with data that supports the conclusions. Experiments must have been conducted rigorously, with appropriate controls, replication, and sample sizes. The conclusions must be drawn appropriately based on the data presented. 

Reviewer #1: Yes

Reviewer #2: Yes

Reviewer #3: Yes

**********

2. Has the statistical analysis been performed appropriately and rigorously? 

Reviewer #1: Yes

Reviewer #2: Yes

Reviewer #3: N/A

**********

3. Have the authors made all data underlying the findings in their manuscript fully available?

The PLOS Data policy requires authors to make all data underlying the findings described in their manuscript fully available without restriction, with rare exception (please refer to the Data Availability Statement in the manuscript PDF file). The data should be provided as part of the manuscript or its supporting information, or deposited to a public repository. For example, in addition to summary statistics, the data points behind means, medians and variance measures should be available. If there are restrictions on publicly sharing data—e.g. participant privacy or use of data from a third party—those must be specified.

Reviewer #1: Yes

Reviewer #2: No

Reviewer #3: No

**********

4. Is the manuscript presented in an intelligible fashion and written in standard English?

PLOS ONE does not copyedit accepted manuscripts, so the language in submitted articles must be clear, correct, and unambiguous. Any typographical or grammatical errors should be corrected at revision, so please note any specific errors here.

Reviewer #1: Yes

Reviewer #2: Yes

Reviewer #3: No

**********

5. Review Comments to the Author

Please use the space provided to explain your answers to the questions above. You may also include additional comments for the author, including concerns about dual publication, research ethics, or publication ethics. (Please upload your review as an attachment if it exceeds 20,000 characters)

Reviewer #1: This work includes interesting findings. The authors have investigated the polymorphisms and their possible implications, with particular investigative focus on their associations with mastitis resistance, serum C3 level, complement hemolytic activity (CH50 and ACH50), and milk production traits, Bacterial counts of serum antibacterial activities were also completed to verify the effect of SNPs on resistance to mastitis pathogens.

Comments:

There are too many paragraphs in text, some of paragraphs could be merged. Conclusion need to be rewritten. The manuscript requires English polishing.

The results of gene expression and antibacterial activity have to be summarized and displayed in abstract.

The symbols of significance are missing in Fig 3 and Fig 4. In Fig 3, does the error bar meas SD or SE? In Fig 4, group name should to be consistent with text in line 391.

Line 404: ATG(Met)>ACG(Thr). The loci are at position 19th suggest be changed to ATG (Met) > ACG (Thr). The loci are at position 19 th.

Line 489-491: suggest to add the reference.

Line 509:  Janssen B. J. et al, 2005; Sreis E. et al, 2006 suggest be changed to Janssen et al, 2005; Sreis et al, 2006.

Reviewer #2: The article entitled Complement component 3 haplotypes influence serum complement activity and milk production traits in Chinese Holstein cattle is a well written manuscript that depicts an interesting research.

However, the file that was presented to me for review did not contain tables mentioned in the manuscript, therefore I was not able to verify the data described in the text.

Line 165 – please provide information on what breeds were these cattle?

Line 287-8 – this belongs to the introduction.

L 397 – 402 - this belongs to the introduction.

L 405-407 – please add reference regarding the C-terminal role.

It would be good to finish the article with a clear conclusions that would also show possible benefits of the findings. AS it is the second to last paragraph is more of a conclusion than the last paragraph.

Reviewer #3: Dear Authors,

The authors results showed that complement component 3 haplotypes influence serum complements activity and milk production traits in Chinese Holstein cattle.

However, I think that it is necessary to strengthen the reliability of the result by adding as much information as possible for readers.

Major comments:

1. I recommend a flow diagram of the number of cattle included and clarify the number of samples you used for each analysis. Then clarify the content of the discussion section. I cannot understand relative with results of each analysis and discussion.

2. The authors should be more clearly stated in the discussion section.

3. I think your research is good, but your sample numbers are not clear enough for most readers to understand. I strongly recommend the authors check and correct their manuscript.

4. I would like you to clearly describe in the discussion how the clarification of this research will be useful in agriculture practice.

5. I would like you to recommend include a photo of the three Chinese Holstein cattle species and describe

their characteristics in the Methods section.

6. Also, please specify the relationship between the characteristics of the three Chinese Holstein cattle species and your results.

7. The authors should check the calculation method to your test.

8. Also, please revise some English texts, again.

**********

6. PLOS authors have the option to publish the peer review history of their article (what does this mean?). If published, this will include your full peer review and any attached files.

If you choose “no”, your identity will remain anonymous but your review may still be made public.

**Do you want your identity to be public for this peer review?** For information about this choice, including consent withdrawal, please see our Privacy Policy.

Reviewer #1: No

Reviewer #2: No

Reviewer #3: No

[NOTE: If reviewer comments were submitted as an attachment file, they will be attached to this email and accessible via the submission site. Please log into your account, locate the manuscript record, and check for the action link "View Attachments". If this link does not appear, there are no attachment files.]

While revising your submission, please upload your figure files to the Preflight Analysis and Conversion Engine (PACE) digital diagnostic tool, https://pacev2.apexcovantage.com/. PACE helps ensure that figures meet PLOS requirements. To use PACE, you must first register as a user. Registration is free. Then, login and navigate to the UPLOAD tab, where you will find detailed instructions on how to use the tool. If you encounter any issues or have any questions when using PACE, please email PLOS at figures@plos.org. Please note that Supporting Information files do not need this step.

# Author comment: Author response to Decision Letter 0

**DOI:** 10.1371/journal.pone.0268959.r002

2 Mar 2022

Dear Editor,

We are very thankful for the reviews which gave us an opportunity to improve our manuscript. Our manuscript has been revised according to the review’s comments.

We hope that after this revision it will achieve the publication requirements of PLoS ONE Journal. Below there are our answers (in blue) for all remarks issued by reviewers.

Finally, we restructured the paper to follow PLoS ONE format.

The following are our point-to-point responses.

Acknowledgement

Our deepest gratitude goes to the anonymous reviewer for their careful work and thoughtful suggestions that improved the manuscript substantially.

Changfa Wang

Ph.D., Professor

College of Agronomy, Liaocheng University, 

No.1 Hunan Road, Dongchangfu District, Liaocheng City, Shandong Province, P. R. China, Zip code: 252059

 

Reviewer 1:

Q: There are too many paragraphs in text, some of paragraphs could be merged. Conclusion need to be rewritten. The manuscript requires English polishing.

A: Thanks for your suggestions. We have already polished English and modified manuscript.

Q: The results of gene expression and antibacterial activity have to be summarized and displayed in abstract.

A: Thanks for your suggestions. We have already modified the manuscript and summarized gene expression and antibacterial activity in abstract.

Q: The symbols of significance are missing in Fig 3 and Fig 4. In Fig 3, does the error bar means SD or SE? In Fig 4, group name should to be consistent with text in line 391.

A: Thanks for your suggestions. Data are means ± standard deviation in Fig. 3. Additionally, the group name has changed and consistent with text in Fig. 4.

Q: Line 404: ATG (Met)>ACG (Thr). The loci are at position 19th suggest be changed to ATG (Met) > ACG (Thr). The loci are at position 19th.

A: Thanks for your suggestions. We have already corrected this error.

Q: Line 489-491: suggest to add the reference.

A: Thanks for your suggestions. We have already added the reference.

Q: Line 509: Janssen B. J. et al, 2005; Sreis E. et al, 2006 suggest be changed to Janssen et al, 2005; Sreis et al, 2006.

A: Thanks for your suggestions. We have already changed the format.

 

Reviewer 2:

Q: Line 165 please provide information on what breeds were these cattle?

A: Thanks for your suggestions. The information of breeds on these cattle we provided in introduction part. “The animals used in this study were selected from three common Chinese bovine breeds, including Chinese Holstein (CH; n=821), Luxi Yellow (LX; n=106), and Bohai Black (BH; n=25)” (now line 93-95).

Q: Line 287-8 this belongs to the introduction.

A: Thanks for your suggestions. We have already moved this part to introduction.

Q: L 397-402 this belongs to the introduction.

A: Thanks for your suggestions. We have already moved this part to introduction.

Q: L 405-407 please add reference regarding the C-terminal role.

A: Thanks for your suggestions. We have already added the reference.

Q: It would be good to finish the article with a clear conclusion that would also show possible benefits of the findings. AS it is the second to last paragraph is more of a conclusion than the last paragraph.

A: Thanks for your suggestions. We have already modified the conclusion and discussion part.

 

Reviewer 3:

Q: I recommend a flow diagram of the number of cattle included and clarify the number of samples you used for each analysis. Then clarify the content of the discussion section. I cannot understand relative with results of each analysis and discussion.

A: Thanks for your suggestions. We have already modified the discussion section.

Q: The authors should be more clearly stated in the discussion section.

A: Thanks for your suggestions. We have already modified the discussion section.

Q: I think your research is good, but your sample numbers are not clear enough for most readers to understand. I strongly recommend the authors check and correct their manuscript.

A: Thanks for your suggestions. We have already polished English and modified manuscript.

Q: I would like you to clearly describe in the discussion how the clarification of this research will be useful in agriculture practice.

A: Thanks for your suggestions. We have already modified the discussion section.

Q: I would like you to recommend include a photo of the three Chinese Holstein cattle species and describe their characteristics in the Methods section.

A: Thanks for your suggestions. 

Chinese Holstein Cattle

Luxi Yellow cattle

Bohai Black cattle

Q: Also, please specify the relationship between the characteristics of the three Chinese Holstein cattle species and your results.

A: Thanks for your suggestions. Chinese Holstein Cattle is derived from grading cross breeding and selection between Chinese native cow and purebred Holstein bull introduced in China. The frequency of mastitis in this breed is about 38%–50% [17]. The Luxi Yellow cattle and Bohai Black cattle are two of the representative indigenous bovine (Bos taurus) breeds in China, which have been bred as beef and draft dual-purpose cattle for thousands of years because of their disease resistance and ability to endure unfavorable feeding conditions. 

Q: The authors should check the calculation method to your test.

A: Thanks for your suggestions. We have already checked the calculation method.

Q: Also, please revise some English texts, again.

A: Thanks for your suggestions. We have already polished English and modified manuscript.

Attachment Submitted filename: 3.2_Reponse Letter.docx Click here for additional data file.

# Peer review: Decision Letter 1

**DOI:** 10.1371/journal.pone.0268959.r003

**Authors:** Tomoyoshi Komiyama

24 Mar 2022

PONE-D-21-26730R1Complement component 3 haplotypes influence serum complement activity and milk production traits in Chinese Holstein cattlePLOS ONE

Dear Dr. Wang,

Thank you for submitting your manuscript to PLOS ONE. After careful consideration, we feel that it has merit but does not fully meet PLOS ONE’s publication criteria as it currently stands. Therefore, we invite you to submit a revised version of the manuscript that addresses the points raised during the review process.

Please submit your revised manuscript by May 08 2022 11:59PM. If you will need more time than this to complete your revisions, please reply to this message or contact the journal office at plosone@plos.org. When you're ready to submit your revision, log on to https://www.editorialmanager.com/pone/ and select the 'Submissions Needing Revision' folder to locate your manuscript file.

Please include the following items when submitting your revised manuscript:

- A rebuttal letter that responds to each point raised by the academic editor and reviewer(s). You should upload this letter as a separate file labeled 'Response to Reviewers'.
- A marked-up copy of your manuscript that highlights changes made to the original version. You should upload this as a separate file labeled 'Revised Manuscript with Track Changes'.
- An unmarked version of your revised paper without tracked changes. You should upload this as a separate file labeled 'Manuscript'.

If you would like to make changes to your financial disclosure, please include your updated statement in your cover letter. Guidelines for resubmitting your figure files are available below the reviewer comments at the end of this letter.

If applicable, we recommend that you deposit your laboratory protocols in protocols.io to enhance the reproducibility of your results. Protocols.io assigns your protocol its own identifier (DOI) so that it can be cited independently in the future. For instructions see: https://journals.plos.org/plosone/s/submission-guidelines#loc-laboratory-protocols. Additionally, PLOS ONE offers an option for publishing peer-reviewed Lab Protocol articles, which describe protocols hosted on protocols.io. Read more information on sharing protocols at https://plos.org/protocols?utm_medium=editorial-email&utm_source=authorletters&utm_campaign=protocols.

We look forward to receiving your revised manuscript.

Kind regards,

Tomoyoshi Komiyama, Ph.D

Academic Editor

PLOS ONE

Journal Requirements:

Please review your reference list to ensure that it is complete and correct. If you have cited papers that have been retracted, please include the rationale for doing so in the manuscript text, or remove these references and replace them with relevant current references. Any changes to the reference list should be mentioned in the rebuttal letter that accompanies your revised manuscript. If you need to cite a retracted article, indicate the article’s retracted status in the References list and also include a citation and full reference for the retraction notice.

Additional Editor Comments (if provided):

Dear authors,

Thank you for submitting your revised manuscript.

I think it is easier to understand than the previous manuscript.

However, it received some additional questions from the reviewer.

Please answer these questions.

Tomoyoshi Komiyama

[Note: HTML markup is below. Please do not edit.]

Reviewers' comments:

Reviewer's Responses to Questions

**Comments to the Author**

1. If the authors have adequately addressed your comments raised in a previous round of review and you feel that this manuscript is now acceptable for publication, you may indicate that here to bypass the “Comments to the Author” section, enter your conflict of interest statement in the “Confidential to Editor” section, and submit your "Accept" recommendation.

Reviewer #1: All comments have been addressed

Reviewer #2: (No Response)

Reviewer #3: All comments have been addressed

**********

2. Is the manuscript technically sound, and do the data support the conclusions?

The manuscript must describe a technically sound piece of scientific research with data that supports the conclusions. Experiments must have been conducted rigorously, with appropriate controls, replication, and sample sizes. The conclusions must be drawn appropriately based on the data presented. 

Reviewer #1: Yes

Reviewer #2: Yes

Reviewer #3: Yes

**********

3. Has the statistical analysis been performed appropriately and rigorously? 

Reviewer #1: Yes

Reviewer #2: Yes

Reviewer #3: Yes

**********

4. Have the authors made all data underlying the findings in their manuscript fully available?

The PLOS Data policy requires authors to make all data underlying the findings described in their manuscript fully available without restriction, with rare exception (please refer to the Data Availability Statement in the manuscript PDF file). The data should be provided as part of the manuscript or its supporting information, or deposited to a public repository. For example, in addition to summary statistics, the data points behind means, medians and variance measures should be available. If there are restrictions on publicly sharing data—e.g. participant privacy or use of data from a third party—those must be specified.

Reviewer #1: Yes

Reviewer #2: Yes

Reviewer #3: Yes

**********

5. Is the manuscript presented in an intelligible fashion and written in standard English?

PLOS ONE does not copyedit accepted manuscripts, so the language in submitted articles must be clear, correct, and unambiguous. Any typographical or grammatical errors should be corrected at revision, so please note any specific errors here.

Reviewer #1: Yes

Reviewer #2: Yes

Reviewer #3: Yes

**********

6. Review Comments to the Author

Please use the space provided to explain your answers to the questions above. You may also include additional comments for the author, including concerns about dual publication, research ethics, or publication ethics. (Please upload your review as an attachment if it exceeds 20,000 characters)

Reviewer #1: (No Response)

Reviewer #2: Thera are still some minor changes that can be made to the language.

Discussion – it seems that some paragraphs in Discussion contain only the description of results and then a sentence form the authors providing a conclusion or a an attempt to explain these results. I feel that these paragraph could use also referencing to other studies.

The last paragraph of the manuscript should have a clear conclusions. Please add such a paragraph, as it is the last paragraph (“For humans, homozygous deficiency at the C3 locus…..”) is still a discussion. The paragraph before is more of a conclusion. A reader would like to know what is your thoughts and summary of the research, potential use – and they would typically look at the last paragraph.

Reviewer #3: Thank you for submitting your revised manuscript.

The authors revised the sentences according to my comments.

Therefore, I have no further questions.

**********

7. PLOS authors have the option to publish the peer review history of their article (what does this mean?). If published, this will include your full peer review and any attached files.

If you choose “no”, your identity will remain anonymous but your review may still be made public.

**Do you want your identity to be public for this peer review?** For information about this choice, including consent withdrawal, please see our Privacy Policy.

Reviewer #1: No

Reviewer #2: No

Reviewer #3: No

[NOTE: If reviewer comments were submitted as an attachment file, they will be attached to this email and accessible via the submission site. Please log into your account, locate the manuscript record, and check for the action link "View Attachments". If this link does not appear, there are no attachment files.]

While revising your submission, please upload your figure files to the Preflight Analysis and Conversion Engine (PACE) digital diagnostic tool, https://pacev2.apexcovantage.com/. PACE helps ensure that figures meet PLOS requirements. To use PACE, you must first register as a user. Registration is free. Then, login and navigate to the UPLOAD tab, where you will find detailed instructions on how to use the tool. If you encounter any issues or have any questions when using PACE, please email PLOS at figures@plos.org. Please note that Supporting Information files do not need this step.

# Author comment: Author response to Decision Letter 1

**DOI:** 10.1371/journal.pone.0268959.r004

27 Apr 2022

Reviewer 1:

No questions

Reviewer 2:

Q: Thera are still some minor changes that can be made to the language.

A: Thanks for your suggestions. We have already modified the manuscript.

Q: The last paragraph of the manuscript should have a clear conclusion. Please add such a paragraph, as it is the last paragraph (“For humans, homozygous deficiency at the C3 locus….”) is still a discussion. The paragraph before is more of a conclusion. A reader would like to know what is your thoughts and summary of the research, potential use – and they would typically look at the last paragraph.

A: Thanks for your suggestions. We have already changed the discussion part and added the last paragraph.

Reviewer 3:

No questions

Attachment Submitted filename: 4.17_Reponse Letter.docx Click here for additional data file.

# Editor report: Decision Letter 2

**DOI:** 10.1371/journal.pone.0268959.r005

**Authors:** Tomoyoshi Komiyama

12 May 2022

Complement component 3 haplotypes influence serum complement activity and milk production traits in Chinese Holstein cattle

PONE-D-21-26730R2

Dear Dr. Wang,

We’re pleased to inform you that your manuscript has been judged scientifically suitable for publication and will be formally accepted for publication once it meets all outstanding technical requirements.

Within one week, you’ll receive an e-mail detailing the required amendments. When these have been addressed, you’ll receive a formal acceptance letter and your manuscript will be scheduled for publication.

An invoice for payment will follow shortly after the formal acceptance. To ensure an efficient process, please log into Editorial Manager at http://www.editorialmanager.com/pone/, click the 'Update My Information' link at the top of the page, and double check that your user information is up-to-date. If you have any billing related questions, please contact our Author Billing department directly at authorbilling@plos.org.

If your institution or institutions have a press office, please notify them about your upcoming paper to help maximize its impact. If they’ll be preparing press materials, please inform our press team as soon as possible -- no later than 48 hours after receiving the formal acceptance. Your manuscript will remain under strict press embargo until 2 pm Eastern Time on the date of publication. For more information, please contact onepress@plos.org.

Kind regards,

Tomoyoshi Komiyama, Ph.D

Academic Editor

PLOS ONE

Additional Editor Comments (optional):

Dear authors,

Thank you for submitting your revised manuscript.

I think it was much easier to understand than the original manuscript.

I am satisfied with the responses and the edits, I am happy to accept this manuscript.

The authors have replied to my remaining questions satisfactorily from three reviewers.

Therefore, I have no further comments to make, all of my previous concerns were adequately addressed.

This manuscript will be satiating the reader's interest.

Tomoyoshi Komiyama

Reviewers' comments:

# Editor report: Acceptance letter

**DOI:** 10.1371/journal.pone.0268959.r006

**Authors:** Tomoyoshi Komiyama

7 Jun 2022

PONE-D-21-26730R2 

Complement component 3 haplotypes influence serum complement activity and milk production traits in Chinese Holstein cattle 

Dear Dr. Wang:

I'm pleased to inform you that your manuscript has been deemed suitable for publication in PLOS ONE. Congratulations! Your manuscript is now with our production department. 

If your institution or institutions have a press office, please let them know about your upcoming paper now to help maximize its impact. If they'll be preparing press materials, please inform our press team within the next 48 hours. Your manuscript will remain under strict press embargo until 2 pm Eastern Time on the date of publication. For more information please contact onepress@plos.org.

If we can help with anything else, please email us at plosone@plos.org. 

Thank you for submitting your work to PLOS ONE and supporting open access. 

Kind regards, 

PLOS ONE Editorial Office Staff

on behalf of

Dr. Tomoyoshi Komiyama 

Academic Editor

PLOS ONE
